# Three-Dimensional COF with “the” Topology as Enzyme Host: Comparative Insights into Activity, Stability, and Reusability in Surface versus Pore Immobilization Strategies

**DOI:** 10.1021/acsmaterialsau.5c00098

**Published:** 2025-10-02

**Authors:** Kohki Sasaki, Tsukasa Irie, Jin Sakai, Yu Zhao, Mika Nozaki, Tokuhisa Kawawaki, Saikat Das, Teng Ben, Yuichi Negishi

**Affiliations:** † Institute of Multidisciplinary Research for Advanced Materials, 13101 Tohoku University, 2-1-1 Katahira, Aoba-ku, Sendai 980-8577, Japan; ‡ Department of Applied Chemistry, Faculty of Science, 26413 Tokyo University of Science, Kagurazaka, Shinjuku-ku, Tokyo 162-8601, Japan; § Zhejiang Engineering Laboratory for Green Syntheses and Applications of Fluorine-Containing Specialty Chemicals, Institute of Advanced Fluorine-Containing Materials, 66344 Zhejiang Normal University, 321004 Jinhua, China

**Keywords:** covalent organic framework, lipase immobilization, catalytic activity, stability, recyclability

## Abstract

Three-dimensional (3D) covalent organic frameworks (COFs) with high connectivity provide structurally rigid yet finely tunable scaffolds that enable precise enzyme immobilization by offering well-defined binding sites and framework stability—key to balancing substrate accessibility with enzyme protection, both critical for efficient biocatalysis. In this work, we investigate the effects of enzyme localization—surface anchoring versus pore entrapment—on catalytic performance by employing two structurally distinct 3D COFs, TUS-39 and TUS-64, as host matrices. We herein report the designed synthesis of TUS-39, a new (8,3)-connected COF featuring **the** topology and microporous structure (0.9 nm) through dynamic imine condensation between a *D*_2h_-symmetric tetragonal prism node and a *D*_3h_-symmetric planar triangular linker. This architecture enabled efficient surface anchoring of amano lipase PS, resulting in remarkably high catalytic activity and reusability in the kinetic resolution of racemic (*R*,*S*)-1-phenylethanol via transesterification. In contrast, the mesoporous (4.7 nm) COF TUS-64 facilitated encapsulation of the enzyme within its pore channels, affording enhanced stability under harsh chemical and thermal environments. The comparative study reveals that surface immobilization on the tightly connected microporous network of TUS-39 enhances substrate accessibility and catalytic conversion rate, while the internal confinement within the larger mesopores of TUS-64 protects the biocatalyst from denaturation and degradation, albeit with a modest trade-off in catalytic efficiency. These findings underscore the critical interplay between surface characteristics, pore metrics, and enzyme localization in dictating the overall efficiency, resilience, and recyclability of COF-supported biocatalysts.

## Graphical abstract

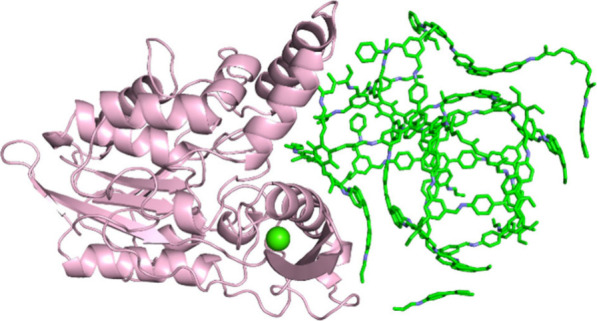

## 1 Introduction

Covalent organic frameworks (COFs) are an emerging class of crystalline porous polymers constructed through the reticulation of organic building blocks via strong covalent interactions.^1–17^ Owing to their periodicity, high surface area, versatile chemical functionality, and robust structural integrity, COFs have garnered substantial attention for applications spanning gas storage,^18,19^ separation,^20,21^ catalysis,^22,23^ energy conversion,^24,25^ and environmental remediation.^26,27^ While two-dimensional (2D) COFs—characterized by layered structures with π–π stacking—have been widely studied due to their relatively facile synthesis and structural predictability, three-dimensional (3D) COFs offer a transformative leap in framework design. Unlike their 2D counterparts, 3D COFs possess spatially extended networks with interconnected pore channels that enable isotropic mass transport, enhanced mechanical rigidity, and increased accessibility of active sites, thereby expanding their functional utility in fields requiring efficient molecular diffusion, such as heterogeneous catalysis and membrane separation.^28,29^

Among 3D COFs, those with high connectivity—built from multitopic monomers such as hexatopic,^30–35^ octatopic,^36–42^ dodecatopic,^43,44^ or even hexadecatopic^45^ linkers—represent a pinnacle of synthetic and structural complexity. These highly connected architectures exhibit intricate topologies, ultrahigh surface areas, and enhanced framework robustness, providing unique advantages in terms of structural integrity under stress and adaptability to multifunctional applications.^46^ The uniform, rigid scaffolds of such COFs can facilitate cooperative interactions across the network, offering synergistic effects in catalysis and sorption processes. However, the realization of these sophisticated frameworks remains a formidable challenge. The construction of highly connected 3D COFs demands precise control over the geometry and reactivity of the building units, as the formation of complex topologies often encounters kinetic hindrances, structural disorder, and difficulties in error correction during framework growth. Moreover, the synthesis of high-denticity organic linkers—especially without the templating or stabilizing role of metal ions—requires multistep organic synthesis and careful consideration of solubility, symmetry, and functional group compatibility. Despite these obstacles, recent breakthroughs have demonstrated the feasibility of designing and synthesizing highly connected 3D COFs, paving the way for a new era of reticular materials that combine structural elegance with unparalleled functionality.

Enzymes are nature’s precision catalysts, renowned for their remarkable selectivity, catalytic efficiency, and ability to mediate complex chemical transformations under benign conditions. Their capacity to operate with high specificity minimizes undesired side reactions, thereby affording cleaner processes with enhanced yields—attributes that are invaluable in pharmaceutical synthesis, fine chemical production, and green chemistry applications.^47,48^ However, the practical use of enzymes in industrial processes is often hampered by intrinsic limitations, such as sensitivity to temperature, pH fluctuations, organic solvents, and susceptibility to denaturation or inhibition under nonphysiological environments.^49^ To circumvent these constraints, immobilization of enzymes onto solid supports has emerged as an effective strategy to not only stabilize the enzymes under harsh conditions but also enable their facile recovery and reuse, thus enhancing process economics and sustainability. Immobilized enzymes typically exhibit greater thermal and operational stability, improved resistance to degradation, and prolonged catalytic lifetimes, especially when appropriately matched with a supporting matrix.^50^

The choice of support material is critical, as it dictates the interaction between the enzyme and its microenvironment, influencing both catalytic activity and durability. Traditional sol–gel matrices, based on silica or hybrid siloxane materials, offer mild encapsulation conditions and good mechanical stability; however, they often suffer from pore shrinkage, mass transfer limitations, and leaching of the enzyme, which reduce long-term usability.^51,52^ Hydrogels, known for their high water content and biocompatibility, provide an ideal aqueous microenvironment for enzyme functionality, but their mechanical fragility, diffusional limitations, and susceptibility to microbial contamination limit their deployment in rigorous industrial settings.^53^ Inorganic supports such as silica nanoparticles and mesoporous oxides are widely employed due to their large surface area and structural robustness, yet they frequently lack chemical tunability and may undergo structural degradation or surface fouling over repeated cycles.^54^

Metal–organic frameworks (MOFs), with their crystalline, tunable architectures and high porosity, have recently garnered attention as enzyme carriers. They can offer mild encapsulation and protective environments; however, challenges such as framework instability in aqueous or acidic media and difficulties in achieving controlled enzyme orientation persist.^55^ In this context, COFs have emerged as a next-generation class of supports, combining the structural precision of MOFs with superior chemical and thermal resilience. COFs, constructed entirely from light elements and robust covalent bonds, present highly ordered structures, adjustable pore dimensions, and exceptional stability— features that collectively foster an enzyme-friendly microenvironment, thereby boosting catalytic activity and prolonging functional lifespan.^56–58^ In particular, high-connectivity 3D COFs might offer interconnected porous networks that are beneficial for enzyme loading, substrate diffusion, and robust framework integrity under operational conditions.^59^

In this contribution, we present the successful synthesis of a new microporous 3-periodic (8,3)-connected COF, designated as TUS-39, with **the** net (Scheme 1). The well-defined microporous structure of TUS-39 endows it with a high surface area, enabling substantial enzyme loading and fostering efficient enzyme–substrate interactions. To further examine the effect of pore dimensionality on biocatalyst behavior, we also synthesized a mesoporous [6 + 4] COF, TUS-64,^32^ which possesses robust 3D connectivity but offers larger internal cavities. We explored two distinct immobilization scenarios: adsorption of enzymes onto the external surface of TUS-39 and encapsulation within the internal pores of TUS-64. Remarkably, surface-immobilized enzymes on TUS-39 demonstrated superior catalytic conversion and excellent reusability, retaining significant activity across multiple catalytic cycles. Conversely, enzymes entrapped within the mesopores of TUS-64 displayed enhanced thermal and chemical durability, withstanding elevated temperatures and harsh solvent environments—conditions under which free enzymes or surface-bound counterparts typically degrade. These complementary behaviors underscore the fundamental role that COF pore size, connectivity, and immobilization mode in steering enzyme performance. The juxtaposition of TUS-39 and TUS-64 as immobilization matrices highlights a versatile design strategy: surface-bound systems offer superior activity and recyclability, whereas pore-confined systems afford greater structural protection.

**Scheme 1:**
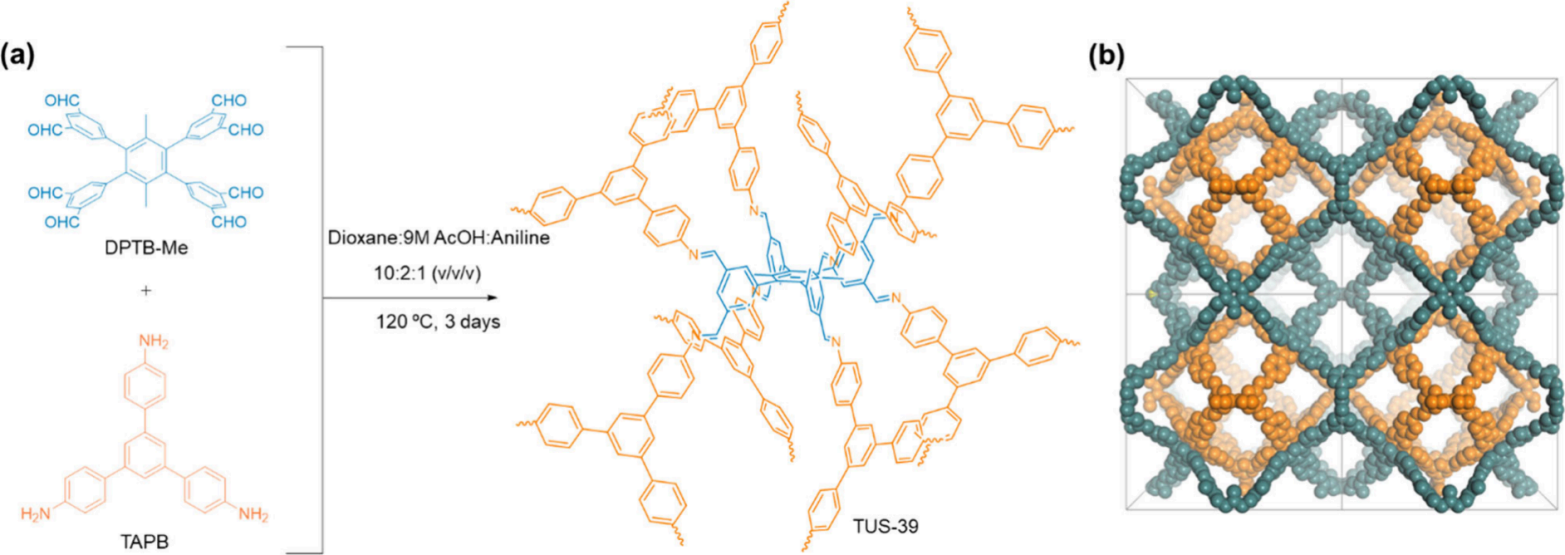
Synthesis Route to 3D COF Featuring “**the**” Topology^a^

a (a) The formation of TUS-39, a 3D COF with **the-c** net topology, arises from a Schiff base reaction between an octatopic tetragonal prism-shaped monomer (DPTB-Me, 3D-*D*_2h_ symmetry) and a planar triangular monomer (TAPB, 2D-*D*_3h_ symmetry). (b) Depiction of the extended network of TUS-39.

## 2 Experimental Section

### 2.1 Synthesis of TUS-39

4′,5′-Bis­(3,5-diformylphenyl)-3′,6′-dimethyl-[1,1′:2′,1″-terphenyl]-3,3″,5,5″-tetracarbaldehyde, DPTB-Me (19.0 mg, 0.03 mmol) and 1,3,5-tris­(4-aminophenyl)­benzene, TAPB (28.1 mg, 0.08 mmol) were loaded into a borosilicate glass tube (8 mm i.d. × 10 mm o.d.), and 1.0 mL of anhydrous 1,4-dioxane was added. The contents were subjected to ultrasonic agitation for ca. 15 min. To the mixture was added 0.2 mL 9 M acetic acid aqueous solution, and sonication was continued for an additional 15 min. Thereafter, 0.1 mL of aniline was added to the mixture, and the system underwent a third round of ultrasonic treatment (ca. 15 min) to attain thorough dispersion. The tube was flash-frozen at 77 K using a liquid nitrogen bath and degassed through three freeze–pump–thaw cycles. Subsequently, the tube was sealed and heated at 120 °C for 72 h. After the reaction was complete, the obtained precipitate was separated by centrifugation and washed repeatedly with THF until the eluate ran clear. After Soxhlet extraction with THF for 24 h, the solid was finally dried in vacuo at 100 °C for 12 h to afford a yellow powder of TUS-39 (44.5 mg, 52% yield). Anal. Calcd for C_624_H_396_N_48_: C: 87.49; H, 4.66; N: 7.85. Found: C: 81.30; H: 5.25; N: 7.53.

### 2.2 Synthesis of TUS-64

TUS-64 was prepared following previously reported procedure.^32^

### 2.3 Loading of Amano Lipase PS on TUS-39/64

An enzyme solution was prepared by dissolving 120.0 mg of lipase PS in 5.0 mL of 0.1 M phosphate buffer solution (pH = 7.0). Separately, 25 mg of TUS-39/64 was dispersed in 5.0 mL of distilled water and subjected to sonication for 15 min to obtain a uniform COF suspension. Thereafter, the enzyme solution and COF suspension were mixed, and the resulting mixture was magnetically stirred for 5 h to facilitate immobilization. After that, the resulting lipase@TUS-39/64 was separated via centrifugation.

### 2.4 Determination of Amano Lipase PS Uptake by TUS-39/64

#### From UV–Vis Spectroscopy

To make the bicinchoninic acid (BCA) working reagent, Protein Assay BCA Reagent A and Protein Assay BCA Reagent B (Cu^2+^ solution) were mixed in a 50:1 volume ratio. Meanwhile, 60 μL aliquots of each standard and 10 times diluted unknown sample were transferred to individual vials. Each vial then received 1.2 mL of freshly prepared working reagent, which was mixed thoroughly. The vials were sealed and heated in a 50 °C oven for 15 min, then allowed to cool to ambient temperature for 30 min. Absorbance values at 561 nm were recorded using UV/vis spectroscopy. The concentration of lipase in unknown sample was determined using a standard curve (Figure S11b) derived from the absorbance of the standards (Figure S11a). Prior to and following a 6-h lipase PS loading process on TUS-39 (or TUS-64), the UV/vis absorbance values of the 10-fold diluted supernatants were recorded as 0.0414921 (0.0401153) and 0.0380831 (0.0281822), respectively, with the values in parentheses indicating those for TUS-64. The equation describing the standard calibration curve is

$$y=3.55016\times 10^{-5}x+6.97212\times 10^{-3},R^{2}=0.9999$$

where *y* refers to the absorbance value, *x* to the lipase concentration expressed in ppm, and *R* to the correlation coefficient.

Using the standard curve and absorbance values multiplied by 10, the calculated lipase concentrations before (*C*_1_) and after (*C*_2_) loading on TUS-39 (or TUS-64) were 11491.00 ppm (11103.18 ppm) and 10530.76 ppm (7741.90 ppm), respectively. From these results, the amount of lipase loaded (mg) and the uptake capacity (mg mg^–1^) were evaluated as follows:

- Loading weight of lipase on TUS-39 (mg)
  = ((*C*_1_ – *C*_2_) × Amount of supernatant (mL))/1000 = ((11491.00–10530.76) × 10/1000 = 9.60 mg
- Lipase uptake capacity of TUS-39 (mg mg^–1^)
  = 9.60 mg/50 mg = 0.19 mg mg^–1^
- Loading weight of lipase on TUS-64 (mg)
  = ((*C*_1_ – *C*_2_) × Amount of supernatant (mL))/1000 = ((11103.18–7741.90) × 10/1000 = 33.61 mg
- Lipase uptake capacity of TUS-64 (mg mg^–1^)
  = 33.61 mg/50 mg = 0.67 mg mg^–1^

#### From Elemental Analysis

The weight percentage of nitrogen element was identified as 0.33%, 7.53%, 6.02%, 6.25% and 2.14% in amano lipase PS, TUS-39, TUS-64, lipase@TUS-39, and lipase@TUS-64, respectively. Based on these values, the lipase uptake capacity was calculated as follows:

- Lipase uptake capacity of TUS-39 (mg mg^–1^)
  = (wt % of nitrogen in COF – wt % of nitrogen in lipase@COF)/(wt % of nitrogen in COF – wt % of nitrogen in lipase)
  = (7.53 – 6.25)/(7.53 – 0.33) = 0.18 mg mg^–1^
- Lipase uptake capacity of TUS-64 (mg mg^–1^)
  = (wt % of nitrogen in COF – wt % of nitrogen in lipase@COF)/(wt % of nitrogen in COF – wt % of nitrogen in lipase)
  = (6.02 – 2.14)/(6.02 – 0.33) = 0.68 mg mg^–1^

## 2.5 Assessment of Enzymatic Activity for Lipase@TUS-39/64

The catalytic performance of lipase immobilized on the TUS-39 and TUS-64 COF supports (lipase@TUS-39/64) was systematically investigated through kinetic resolution of *rac*-1-phenylethanol, employing vinyl acetate as the acylating agent and *n*-hexane as the reaction medium. Experiments were carried out in 10 mL vials equipped with magnetic stirrers. The reaction mixture comprised 1-phenylethanol (60.5 μL, 0.50 mmol), vinyl acetate (120.3 μL, 1.30 mmol), *n*-hexane (2.4 mL), and lipase@TUS-39/64 (5.0 mg). Following the preparation of the reaction mixture, the vials were tightly capped and heated at 50 °C in an oil bath while stirring continuously for the required reaction time. Upon completion of the enzymatic reaction, the catalyst was separated via centrifugation, while the resulting clear supernatant was carefully collected and subjected to high-performance liquid chromatography (HPLC) analysis to quantify the conversion rates based on chromatographic peak areas. Chromatographic separations employed a CHIRAL ART Cellulose-SB column with a mobile phase consisting of *n*-hexane and isopropanol in a 95:5 (v/v) ratio. The column was operated at a constant temperature of 25 °C, with a flow rate of 1.0 mL min^–1^. The detection wavelength was set at 254 nm, and each sample was injected in a volume of 20 μL. The chromatographic run time for each sample varied between 15 and 20 min. To assess the reusability, the catalyst was recovered by centrifugation, subjected to vacuum overnight, and subsequently reused in successive reaction cycles without additional treatment or drying.

## 3 Results and Discussion

### 3.1 Synthesis and Characterization of TUS-39

Through systematic condition screening, the Schiff-base polymerization of DPTB-Me and TAPB (3:8 molar ratio) using 1,4-dioxane as solvent, in the presence of 9 M aqueous acetic acid and aniline, at 120 °C for 3 days, furnished TUS-39 as a yellow crystalline product in satisfactory yield. Spectroscopic signatures consistent with imine bond formation in TUS-39 were observed in both Fourier transform infrared (FT-IR) and solid-state ^13^C cross-polarization magic angle spinning (CP/MAS) NMR analyses. A prominent vibration band at 1623 cm^–1^, assigned to the C=N linkage, was observed in the FT-IR spectral profile of the COF. On the other hand, a notable suppression of the characteristic N–H stretching signals of TAPB at 3434 and 3354 cm^–1^ and the C=O stretching band of DPTB-Me at 1703 cm^–1^ points to the successful progression of imine condensation, confirming the consumption of reactive groups during COF formation (Figure S1). Furthermore, a distinct peak at 159.4 ppm in the ^13^C CP/MAS NMR profile was indicative of the imine carbon (C=N), providing further confirmation of the framework’s formation (Figure S2). The elemental composition of TUS-39, determined as C_624_H_396_N_48_, exhibited close agreement between observed (C: 81.30; H: 5.25; N: 7.53) and calculated (C: 87.49; H, 4.66; N: 7.85) values, affirming the fidelity of the COF synthesis and structure. Scanning electron microscopy (SEM) characterization disclosed a coral-like morphology consisting of nanometer-sized particles clustering together into micrometer-scale aggregates (Figure S3). To assess the material’s thermal integrity, thermogravimetric analysis (TGA) was performed in a nitrogen environment and showed a weight loss of ca. 5% at 287 °C, indicative of moderate stability (Figure S4). To probe chemical robustness, the COF was soaked in different solvents for 24 h, and subsequent PXRD measurements confirmed the preservation of its crystalline framework (Figure S5).

### 3.2 Crystal Structure Analysis

A rigorous structural evaluation of TUS-39 was accomplished through comparative analysis of powder X-ray diffraction (PXRD) data with computationally derived models, followed by structural refinement (Figure 1). Geometry optimization was carried out using the Forcite module within *Materials Studio* 7.0,^60^ which applies classical force-field-based energy minimization techniques to refine the atomic structure. Upon optimization, TUS-39 was found to adopt a 2-fold interpenetrated **the** net, crystallizing in the space group *Im*-3 (No. 204) with unit cell parameters of *a* = *b* = *c* = 34.4451 Å and α = β = γ = 90° (Table S2). Characteristic diffraction signals were observed in the PXRD pattern of TUS-39 at 5.6°, 6.2°, 7.2°, 10.3°, and 11.6° which can be indexed to the (200), (211), (220), (400), and (420) lattice planes, respectively (Figure 1). Refinement of the PXRD data via the Pawley method achieved good fit quality with low residual factors (*R*_p_ = 0.81%, *R*_wp_ = 1.08%). An excellent congruence was observed between the PXRD pattern refined via Pawley fitting (red curve) and the experimentally observed data (black dots), with the difference plot (blue) reflecting only marginal inconsistencies. As a comparison, a noninterpenetrated **the** net crystal model in the *Pm*-3 space group (No. 200) was also established (Figure S10); yet its simulated diffraction pattern was inconsistent with the experimental PXRD (Figure S9). PXRD data combined with computational modeling unambiguously support the assignment of TUS-39 to a 2-fold interpenetrated **the** net topology.

**Figure 1:**
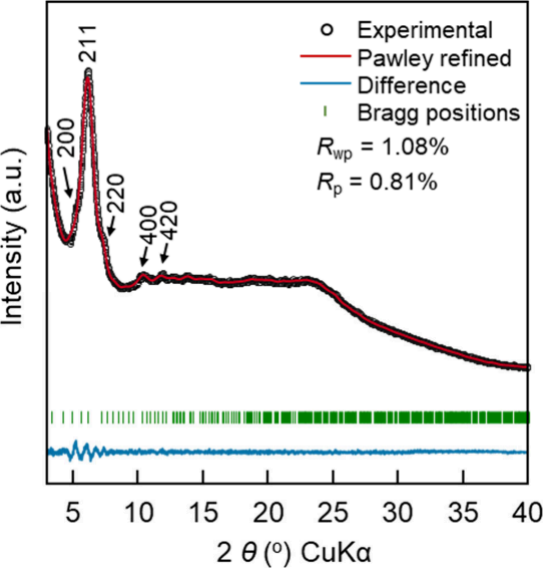
XRD analysis of TUS-39 illustrating the observed pattern (black symbols), Pawley-fitted curve (red trace), difference profile (blue trace) displaying discrepancies between the experimental and refined patterns, and green ticks mark the Bragg reflections.

### 3.3 Assessment of Porosity

Following an 8-h activation at 120 °C, nitrogen sorption studies at 77 K were employed to probe the porosity of TUS-39. As shown in Figure 2a, the observed type I isotherm affirmed its microporous nature, and the Brunauer–Emmett–Teller (BET) surface area was determined to be 1857 m^2^ g^–1^ based on multipoint analysis at low *P*/*P*_0_ (Figure S6). Further interpretation using nonlocal density functional theory (NLDFT) with a slit-pore model indicated a pore volume of 1.661 cm^3^ g^–1^ and a pore size distribution dominated by a single peak at 0.9 nm (Figure 2b), consistent with the predicted dimensions (0.8–0.9 nm) from the structural model (Figure S8).

**Figure 2:**
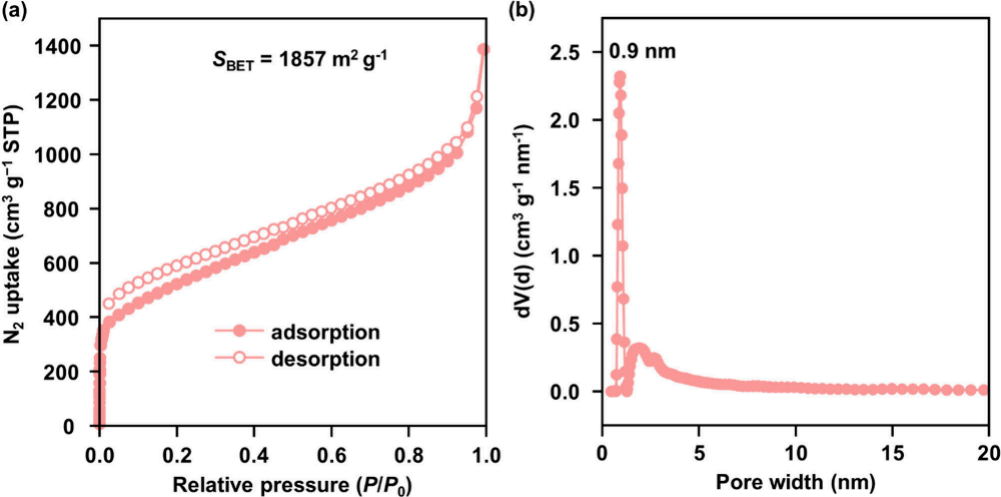
(a) Nitrogen sorption profile of TUS-39 acquired at 77 K and (b) corresponding pore size distribution derived from NLDFT analysis.

### 3.4 Immobilizing Amano Lipase PS onto/into COFs

To harness the structural advantages of highly connected 3D COFs for biocatalysis, we investigated two distinct enzyme immobilization strategies: surface adsorption and internal pore encapsulation. In the first approach, we immobilized amano lipase PS onto the surface of TUS-39. Although the intrinsic pores of TUS-39 cannot accommodate the enzyme, its crystalline and highly connected 3D framework still offers uniformly distributed surface anchoring sites, ensuring controlled orientation and reproducible binding—advantages not achievable with amorphous supports. Given that the molecular dimensions of the lipase (approximately 30 Å × 32 Å × 60 Å)^61^ substantially exceed the pore size of TUS-39 (9 Å), immobilization is rationally attributed to surface adsorption rather than pore confinement. The immobilization was achieved by mixing aqueous suspension of TUS-39 in 5 mL of phosphate buffer (pH 7.0) containing 120 mg of lipase, allowing for noncovalent interactions between the enzyme and the COF’s external surface. The enzyme loading capacity of TUS-39 was quantified using two complementary techniques. The BCA assay, based on UV/vis absorbance at 561 nm, yielded a loading capacity of 0.19 mg of lipase per mg of COF, closely matching the value of 0.18 mg mg^–1^ derived from elemental nitrogen analysis—thus confirming the accuracy and reproducibility of the immobilization process. Notably, the crystallinity of TUS-39 was preserved postimmobilization, as evidenced by the retention of characteristic diffraction peaks in the PXRD pattern of the resulting composite, lipase@TUS-39 (Figure S15). Furthermore, SEM revealed clear morphological evidence of lipase deposition on the nanoscale COF particles, suggesting effective surface coverage by the enzyme (Figure S12). A significant reduction in BET surface area from 1857 m^2^ g^–1^ (pristine TUS-39) to 350 m^2^ g^–1^ (lipase@TUS-39) corroborated the surface adsorption model, implying partial blockage of the external pores by the surface-bound enzyme molecules (Figures S13, S14). This decline not only affirms successful immobilization but also indicates strong surface interactions that restrict access to internal porosity. To confirm that the observed reduction in BET surface area is indeed due to enzyme immobilization and not pore collapse or framework instability, we performed a control experiment in which TUS-39 was soaked for 6 h in buffer without enzyme. The resulting BET surface area was 1846 m^2^ g^–1^ (Figures S16, S17), nearly identical to that of pristine TUS-39 (1857 m^2^ g^–1^), indicating that the framework remains structurally intact.

To further probe the influence of pore dimensionality and internal encapsulation, we synthesized a structurally robust yet mesoporous [6 + 4] 3D COF, TUS-64, possessing significantly larger channels with a pore size of 47 Å—well above the enzyme’s largest dimension. In this case, enzyme immobilization was conducted under similar aqueous buffer conditions, facilitating spontaneous diffusion of the enzyme into the COF’s spacious internal cavities. The enzyme loading capacity for TUS-64 was markedly higher, reaching 0.67 mg mg^–1^ by BCA assay and 0.68 mg mg^–1^ based on nitrogen elemental analysis. These values highlight the benefit of internal volume in accommodating larger enzyme payloads. BET measurements revealed a dramatic surface area decrease from 1542 m^2^ g^–1^ (pristine TUS-64) to 197 m^2^ g^–1^ (lipase@TUS-64), strongly supporting the conclusion that lipase was encapsulated within the COF’s mesoporous channels, rather than merely adsorbed on the surface (Figures S20–S22). The retention of the COF’s original plate-like crystalline morphology postencapsulation, as observed in SEM imaging (Figures S18, S19), further underscored the structural integrity of TUS-64 during the immobilization process. Collectively, these results delineate two distinct immobilization paradigms: surface anchoring on TUS-39 and pore encapsulation in TUS-64.

### 3.5 Lipase-Catalyzed Kinetic Resolution of (*R*,*S*)-1-Phenylethanol

The enantioselective transformation of racemic 1-phenylethanol into its optically pure (*R*)-form holds great promise in the synthesis of pharmaceuticals and fine chemicals. Among the available strategies, enzyme-catalyzed kinetic resolution presents a green, efficient, and cost-effective alternative to traditional asymmetric synthesis.^62^ In this study, we employed immobilized lipase systems on two structurally distinct COFs—TUS-39 and TUS-64—to catalyze the transesterification of (*R*,*S*)-1-phenylethanol using vinyl acetate as the acyl donor in *n*-hexane (Figure 3a). The kinetic resolution of (*R*,*S*)-1-phenylethanol was performed in three independent runs, and the conversion outcomes are presented as mean ± standard deviation. The enantioselectivity of the reaction was confirmed by HPLC analysis, which revealed exclusive conversion of the (*R*)-enantiomer, with no detectable transformation of the (*S*)-form (Figure S23), underscoring the high stereospecificity of the lipase. A comparative evaluation of the catalytic systems revealed striking differences in conversion efficiency. Free lipase exhibited sluggish activity, affording an insignificant 0.03% conversion after 20 min, which only increased to 14.9% after 100 min. In stark contrast, lipase immobilized on TUS-39, where the enzyme resides on the COF surface, displayed a significantly accelerated reaction rate, achieving 28.7% conversion within just 20 min, and reaching the theoretical maximum of 49.8% conversion after 100 min, demonstrating nearly complete kinetic resolution (Figure 3b). These results clearly highlight the advantage of surface accessibility in facilitating rapid substrate–enzyme interactions, which translates into superior catalytic performance. Interestingly, although TUS-64 possesses a larger pore size capable of accommodating the entire lipase molecule within its internal channels, the encapsulated enzyme showed relatively slower kinetics, attaining only 12.5% conversion at 20 min and 41.9% at 40 min. After 100 min, the conversion reached 49.5%, slightly below that of lipase@TUS-39 (Figure 3b). These observations suggest that while encapsulation may enhance enzyme stability, it may also impose diffusional limitations that hinder substrate access to the active site, thereby reducing the overall reaction rate compared to surface-immobilized systems.

**Figure 3:**
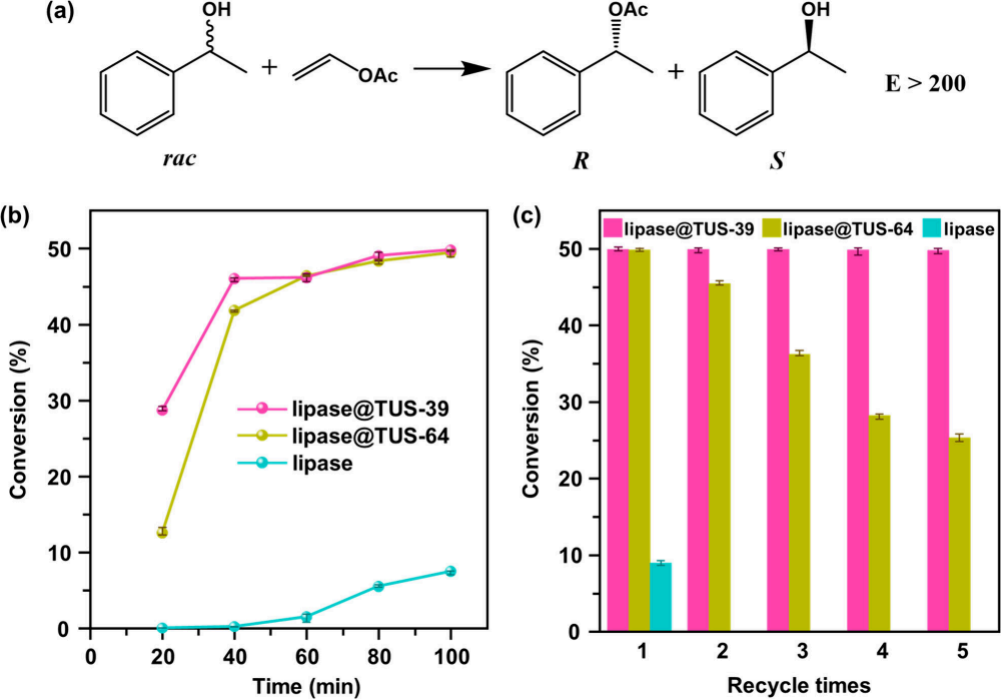
Enantioselective transesterification of (*R*,*S*)-1-phenylethanol using vinyl acetate as the acylating agent and *n*-hexane as the reaction medium: (a) schematic of the transesterification process, (b) comparative catalytic efficiencies of lipase immobilized on TUS-39, TUS-64, and the native enzyme, (c) reusability performance across multiple reaction cycles. Error bars reflect standard deviation of triplicate runs.

Beyond initial activity, catalyst reusability is a vital parameter for evaluating industrial viability. Free lipase, being soluble and structurally vulnerable in aqueous environments, could not be recovered in an active form after a single catalytic cycle. In contrast, lipase@TUS-39 demonstrated exceptional reusability, maintaining 99.7% of its original activity even after five consecutive 6-h reaction cycles (Figure 3c), with no observable loss in catalytic efficiency. This remarkable stability is likely attributed to the structural rigidity and protective environment offered by the COF scaffold, as well as strong noncovalent interactions between the enzyme and the COF surface. Conversely, lipase@TUS-64, although stable initially, exhibited a gradual decline in catalytic performance with repeated use. After five cycles, it retained only 50.7% of its original activity, and a noticeable drop in conversion efficiency was already evident after the first run. Although the enzyme is encapsulated within the pores of TUS-64, several factors could contribute to the gradual loss of activity during recycling. One possible mechanism is pore blockage by substrates, products, or partially denatured enzyme molecules, which can reduce substrate accessibility and lead to decreased activity. In contrast, lipase@TUS-39, being surface-immobilized, benefits from strong noncovalent interactions with the COF surface and unrestricted substrate access. This immobilization mode minimizes structural stress and mass transport limitations, thereby reducing the risk of inactivation. These results further underscore the benefits of surface immobilization over pore encapsulation in maintaining long-term biocatalytic activity, likely due to reduced mass transport barriers and better structural exposure of the enzyme in the former case.

### 3.6 Thermal and Chemical Stability of Immobilized Lipase on TUS-64 and TUS-39

To further evaluate the advantages of COF-assisted immobilization, we next investigated the thermal and chemical stability of lipase when immobilized on TUS-64 and TUS-39. These experiments were aimed at simulating harsh reaction environments and determining how effectively the immobilization strategy can preserve enzymatic activity under such conditions.

The thermal robustness of the immobilized enzymes was first assessed by subjecting the free and immobilized lipase to prolonged exposure at elevated temperatures. As depicted in Figure 4a, native lipase exhibited a dramatic loss of activity upon incubation at 60 °C for 8 h, with a meager 2.8% conversion of (*R*,*S*)-1-phenylethanol. In sharp contrast, lipase@TUS-64 and lipase@TUS-39 demonstrated remarkable resilience, achieving 47.6% and 42.9% conversion, respectively, under the same conditions. When the incubation period was extended to 16 h, lipase@TUS-64 retained nearly full functionality with 47.6% conversion, while lipase@TUS-39 showed a slight decline to 38.5%, still vastly outperforming free lipase, which plummeted to only 1.9% conversion. To probe the stability at more extreme temperatures, the catalytic systems were heated to 120 °C for 8 h. Under these strenuous conditions, lipase@TUS-64 retained an impressive 15.6% conversion, which is significantly higher than the 11.6% achieved by lipase@TUS-39, and far surpasses the negligible 0.3% conversion observed for free lipase. These results firmly establish that the encapsulation of lipase within the spacious internal pores of TUS-64 provides a protective environment that cushions the enzyme against thermal denaturation. The rigid yet porous COF framework likely stabilizes the enzyme’s tertiary structure, shielding it from thermal unfolding and aggregation more effectively than surface immobilization.

**Figure 4:**
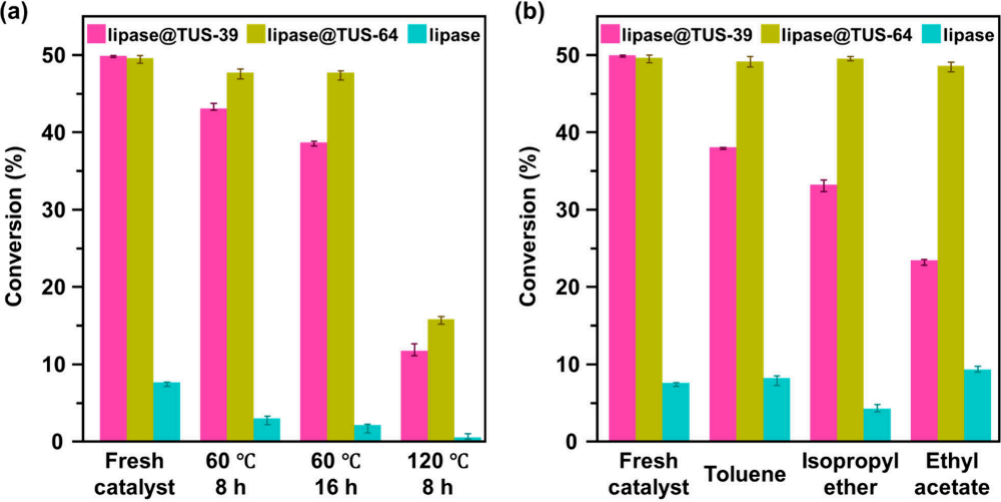
Thermal and chemical stability assessments of immobilized and free lipase during the kinetic resolution of (*R*,*S*)-1-phenylethanol using vinyl acetate and *n*-hexane: (a) comparison of catalytic activity retention after thermal incubation of lipase@TUS-39, lipase@TUS-64, and free lipase, (b) catalytic performance retention after 24 h exposure to various organic solvents.

In parallel, we explored the chemical stability of the immobilized systems by exposing them to common organic solvents for 24 h—a condition that often compromises enzyme integrity. As expected, free lipase suffered considerable deactivation, with the catalytic conversion of (*R*,*S*)-1-phenylethanol dropping to a modest 4.1–9.2%, depending on the solvent used (Figure 4b). Lipase@TUS-39, while more robust than the free enzyme, exhibited only moderate tolerance, retaining 76.0%, 66.3%, and 46.7% of its original activity after treatment with toluene, isopropyl ether, and ethyl acetate, respectively. Strikingly, lipase@TUS-64 emerged as the most chemically durable formulation, maintaining 99.0%, 99.7%, and 97.8% of its initial catalytic activity after identical treatments. This exceptional solvent resistance is attributable to the complete encapsulation of the enzyme within the mesoporous network of TUS-64, which effectively isolates the enzyme from direct exposure to the solvent molecules, preventing structural deformation or active site degradation. The confinement effect, coupled with stabilizing noncovalent interactions within the COF interior, likely contributes to this unprecedented preservation of enzymatic activity. In summary, while TUS-39 enables fast catalysis due to surface accessibility, TUS-64’s encapsulation strategy offers superior protection and longevity, rendering it a highly attractive platform for biocatalytic applications in demanding industrial processes where resilience is paramount.

### 3.7 Enzyme–Framework Interactions

We performed FT-IR and circular dichroism (CD) spectral analyses to probe how the TUS-39 framework influences the secondary and tertiary structure of lipase. In the FT-IR spectra (Figure S24), both free lipase PS and lipase@TUS-39 display a prominent amide I band at ∼1642 cm^–1^, characteristic of C=O stretching vibrations in the peptide backbone, indicating that the overall secondary structure of the enzyme is largely preserved after immobilization. Additionally, lipase and lipase@TUS-39 exhibit a broad absorption band in the 3000–3600 cm^–1^ region, arising from O–H and N–H stretching vibrations associated with hydrogen bonding. This band is absent in pristine TUS-39, suggesting the formation of hydrogen bonds between the enzyme and the COF framework, which supports direct enzyme–framework interactions. Complementary evidence is provided by CD spectroscopy (Figure S25) in the far-UV region (200–250 nm). While pristine TUS-39 shows no detectable signal, free lipase PS exhibits a pronounced negative band around 220 nm with an intensity down to – 30 mdeg mg^–1^, consistent with substantial α-helical content. In contrast, lipase@TUS-39 shows a weaker negative band (−5 mdeg mg^–1^), reflecting a partial reduction of α-helical structure upon immobilization. This attenuation suggests some conformational rearrangement of lipase within the confined framework environment, though the enzyme still retains essential structural features that underpin its catalytic activity.

To gain molecular-level insight into the enzyme–COF interactions, we also performed molecular docking simulations using AutoDock,^63^ a widely recognized platform for predicting ligand–protein interactions. This computational approach allowed us to probe the spatial compatibility and binding affinity between the COF scaffold and the enzyme, elucidating key interaction motifs and energetics that underpin the immobilization process. The docking workflow was meticulously executed using AutoDock Tools 1.5.7 to prepare the ligand and protein structure files. TUS-39 was treated as the ligand (Figure S26), and the crystallographic structure of lipase was used as the receptor (Figure S27). A cubic grid box with dimensions of 220 Å per side was defined to comprehensively cover the protein’s surface and potential binding sites. The grid spacing was set to 0.375 Å, ensuring a fine resolution for the energetic landscape during conformational sampling. For the docking algorithm, the genetic algorithm (GA) was employed, which mimics evolutionary principles to explore a wide conformational space efficiently. A maximum of 100 search conformations was permitted to ensure extensive sampling and accurate binding predictions.

Post-docking, the resultant conformers were ranked based on their predicted binding free energies, and the most energetically favorable structure was selected for further analysis (Figure S28). The optimal binding pose exhibited a docking binding free energy of −13.67 kcal/mol, indicating a strong and thermodynamically favorable interaction between TUS-39 and the lipase. Detailed analysis of the docked complex revealed that six amino acid residues within the binding site engaged in hydrophobic interactions with the framework (Figure 5, Table S1). These residues collectively created a stabilizing hydrophobic environment that promoted effective anchoring of the COF onto the enzyme’s surface.

**Figure 5:**
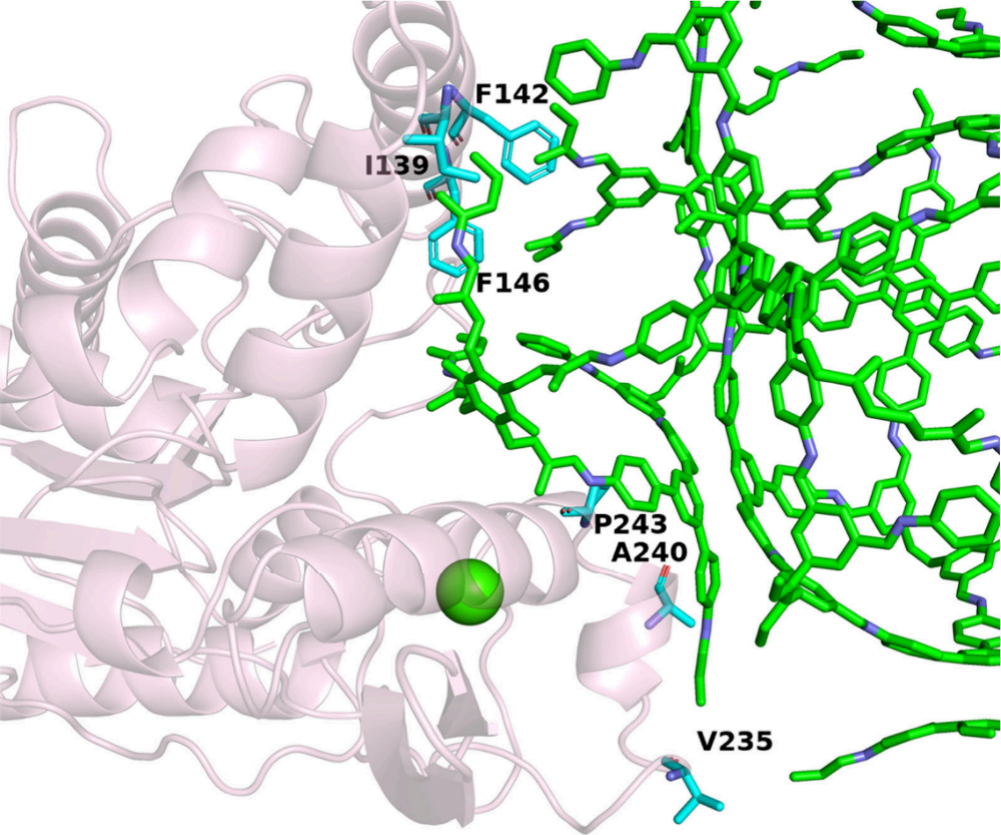
Residue interaction map highlighting local amino acids involved in binding TUS-39 within the active site of amano lipase PS.

Among the noncovalent forces contributing to this binding, van der Waals interactions were identified as the dominant component, playing a critical role in shaping the molecular recognition between the enzyme and the framework. While electrostatic contributions were present, their impact was relatively modest compared to the dispersion-driven forces. This is consistent with the largely nonpolar surface chemistry of TUS-39 and its affinity for hydrophobic protein domains.

The molecular docking results provide compelling evidence that the high surface area and chemical architecture of TUS-39 allow it to intimately engage with the enzyme’s exterior, facilitating stable immobilization without significantly perturbing the protein’s active conformation. These insights reinforce the suitability of TUS-39 as a support matrix for enzyme immobilization and open new avenues for structure-guided design of COFs tailored for biocatalysis.

## 4 Conclusions

This study demonstrates how the structural design of COFs and strategic enzyme localization profoundly influence biocatalytic performance. We introduced a 2-fold interpenetrated **the** net 3D COF developed via the integration of an 8-arm 3D-*D*_2h_ tetragonal prismatic monomer and a 3-arm 2D-*D*_3h_ planar triangular monomer, yielding a highly crystalline framework with large surface area that favors effective surface immobilization of lipase. In the kinetic resolution of racemic (*R*,*S*)-1-phenylethanol, lipase@TUS-39 showed excellent catalytic activity and recyclability. By contrast, TUS-64, a 3D mesoporous COF, allowed internal enzyme encapsulation, offering superior resistance to denaturation and thermal degradation due to pore confinement despite a modest compromise in catalytic efficiency. By comparing surface-anchored and pore-entrapped enzyme systems within TUS-39 and TUS-64, respectively, we reveal the trade-offs between catalytic efficiency and stability. The findings offer valuable guidance for the rational design of COF-based biocatalysts, demonstrating how precise control over pore architecture and tailored enzyme positioning can be strategically leveraged to fine-tune activity, durability, and reusability, thereby advancing the field of framework-supported enzymology.

## Supplementary Material



## Notes

FT-IR spectroscopy, solid-state ^13^C CP-MAS NMR spectroscopy, SEM, TGA, chemical stability, BET surface area plot, structural modeling and XRD analyses, enzyme immobilization, lipase-catalyzed kinetic resolution of (*R*,*S*)-1-phenylethanol, molecular docking simulations, and unit cell information and fractional atomic coordinates (PDF)
