# Supplementary material for: Three-Dimensional COF with “the” Topology as Enzyme Host: Comparative Insights into Activity, Stability, and Reusability in Surface versus Pore Immobilization Strategies
Source: ACS Mater Au. 2025 Oct 2;5(6):991–1000. doi: 10.1021/acsmaterialsau.5c00098 (PMC12616441; doi:10.1021/acsmaterialsau.5c00098)
Supplement: Supplementary file 1 [file mg5c00098_si_001.pdf]

Supporting Information for:

Three-Dimensional COF with "the" Topology as Enzyme Host: Comparative Insights into Activity, Stability, and Reusability in Surface versus Pore Immobilization Strategies

Kohki Sasaki,[a,d] Tsukasa Irie,[a,d] Jin Sakai,[b] Yu Zhao,*[c] Mika Nozaki,[a] Tokuhisa Kawawaki,[a] Saikat Das,*[a] Teng Ben*[c] and Yuichi Negishi*[a]

[a]Institute of Multidisciplinary Research for Advanced Materials, Tohoku University, 2-1-1 Katahira, Aoba-ku, Sendai 980-8577, Japan

[b]Department of Applied Chemistry, Faculty of Science, Tokyo University of Science, Kagurazaka, Shinjuku-ku, Tokyo 162-8601, Japan.

[c]Zhejiang Engineering Laboratory for Green Syntheses and Applications of Fluorine-Containing Specialty Chemicals, Institute of Advanced Fluorine-Containing Materials, Zhejiang Normal University, 321004 Jinhua, China.

[d]These authors contributed equally.

*Email: zhaoyu@zjnu.edu.cn (Y.Z.); das.saikat.c4@tohoku.ac.jp (S.D.); tengben@zjnu.edu.cn (T.B.); yuichi.negishi.a8@tohoku.ac.jp (Y.N.)

Table of Contents

1. General details S3

2. Fourier-Transform Infrared (FT-IR) spectroscopy S5

3. Solid-state ^{13}C CP-MAS Nuclear Magnetic Resonance spectroscopy S6

4. Scanning electron microscopy (SEM) characterization S7

5. Thermogravimetric analysis (TGA) S8

6. Chemical stability S9

7. BET surface area plot S10

8. Structural modeling and X-ray diffraction (XRD) analyses S11

9. Immobilizing amano lipase PS onto/into COFs S13

10. Lipase-catalyzed kinetic resolution of (*R*,*S*)-1-phenylethanol S20

11. FT-IR and circular dichroism (CD) characterization of lipase–TUS-39 interactions S21

12. Molecular docking simulations S23

13. Unit cell information and fractional atomic coordinates S26

14. Supplementary references S27

1. General details

1.1. Reagents. All reagents and solvents, unless noted otherwise, were of analytical grade and used as obtained without any further purification. Anhydrous 1,4-dioxane, acetic acid (AcOH), tetrahydrofuran (THF), toluene, isopropyl ether, ethyl acetate, *n*-hexane, isopropanol, Protein Assay BCA Reagent A, Protein Assay BCA Reagent B, phosphate buffer solution (pH 7.0), vinyl acetate, and (*R*,*S*)-1-phenylethanol, were obtained from FUJIFILM Wako Pure Chemical Corporation. Aniline was obtained from Tokyo Chemical Industry Co., Ltd. Amano lipase PS (from *Burkholderia cepacia*) was obtained from Sigma-Aldrich.

1.2. Instruments.

Powder X-ray diffraction (PXRD): PXRD data were collected on a Rigaku MiniFlex 600 X-ray diffractometer equipped with a Cu Kα radiation source (λ = 0.15406 nm), operated at 40 kV and 15 mA.

Fourier transform infrared (FT-IR) spectroscopy: FT-IR spectra were collected on a JASCO FT/IR-4600 FT-IR spectrometer in the wavenumber range from 4000 to 400 cm^{-1} using the attenuated total reflectance (ATR) method.

Solid-state ^{13}C cross-polarization magic-angle-spinning (CP-MAS) NMR spectroscopy: A Bruker AVANCE NEO 400 MHz NMR spectrometer equipped with a 4-mm VTN probe was employed to obtain solid-state NMR spectra under a sample spinning rate of 10 kHz.

Scanning electron microscopy (SEM): SEM micrographs were recorded using a JEOL JSM-7001F/SHL field emission scanning electron microscope operated at an accelerating voltage of 7 kV. A thin layer of gold was deposited on the sample surface via sputter coating using a Sanyu Electron SC-701Mk Ⅱ ADVANCE sputterer to suppress surface charging.

Nitrogen sorption: Nitrogen physisorption experiments were conducted at 77 K using a Quantachrome Autosorb iQ3 gas sorption analyzer. Prior to measurements, COF samples were activated at 120 °C for 8 hours, while lipase@COF samples were activated at 50 °C for 8 hours using a turbomolecular vacuum pump. The specific surface areas were calculated from nitrogen adsorption data via multipoint Brunauer–Emmett–Teller (BET) method, and pore size distributions were derived from nonlocal density functional theory (NLDFT) on the basis of the model of N_2@77K on carbon with slit pores.

Thermogravimetric analysis (TGA): TGA profiles were recorded on a Bruker TG-DTA2010SA instrument by heating the sample from room temperature to 800 °C under a nitrogen atmosphere at a

ramp rate of 10 °C min^{-1}, with a N_2 flow rate of 50 mL min^{-1}.

Elemental analyses: Elemental analyses of the COFs, lipase and lipase@COFs were carried out with a Elementar vario EL cube elemental analyzer.

UV/Vis spectroscopy: UV/Vis absorption spectra were acquired using a JASCO V-770 spectrophotometer.

1.3. Synthesis of building blocks. 4′,5′-bis(3,5-diformylphenyl)-3′,6′-dimethyl-[1,1′:2′,1″-terphenyl]-3,3″,5,5″-tetracarbaldehyde (DPTB-Me)[1] and 1,3,5-tris(4-aminophenyl)benzene (TAPB)[2,3] were synthesized following previous literature.

2. Fourier-Transform Infrared (FT-IR) spectroscopy

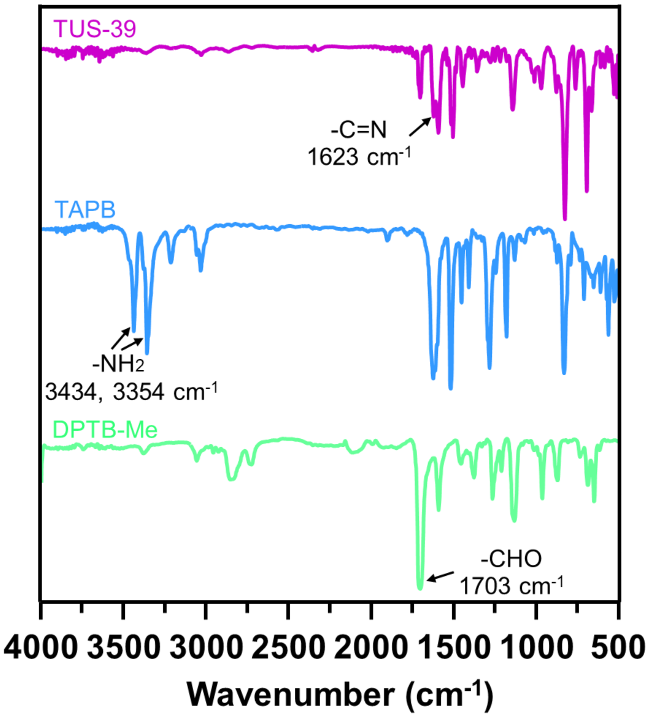

Figure S1. FT-IR spectra of TUS-39 (purple), TAPB (blue), and DPTB-Me (green).

3. Solid-state ^{13}C CP-MAS Nuclear Magnetic Resonance (NMR) spectroscopy

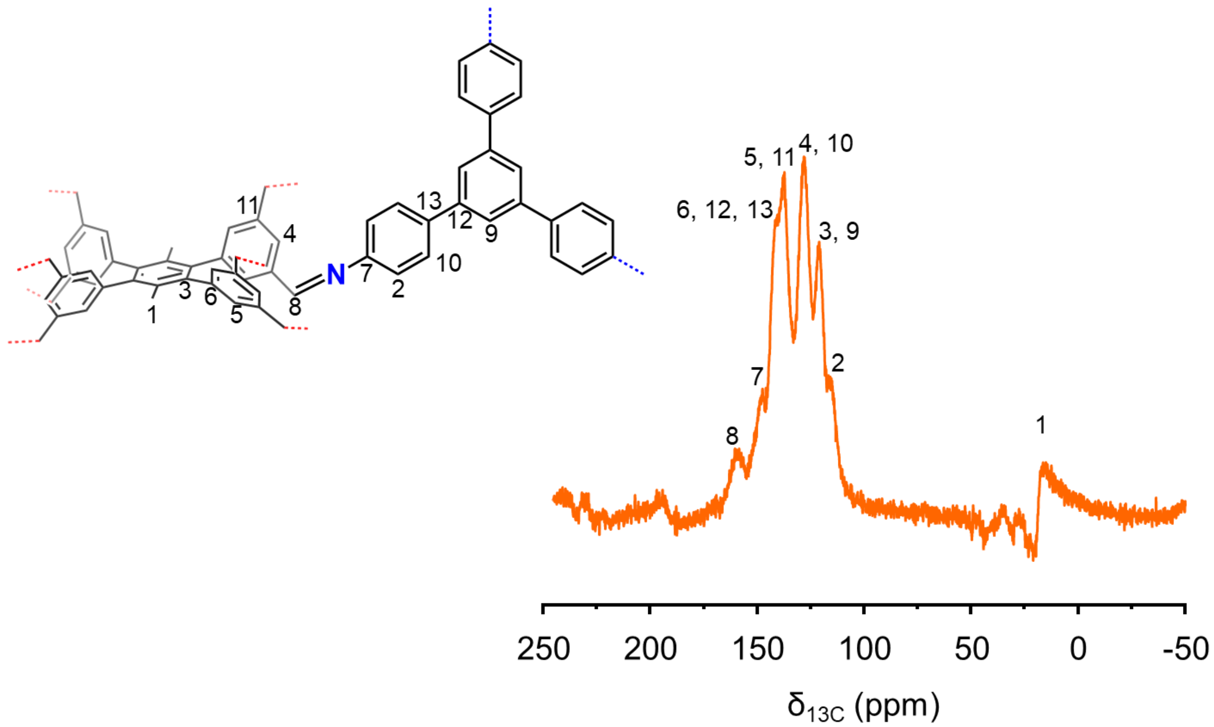

Figure S2. Solid-state ^{13}C CP-MAS NMR spectrum of TUS-39.

4. Scanning electron microscopy (SEM) characterization

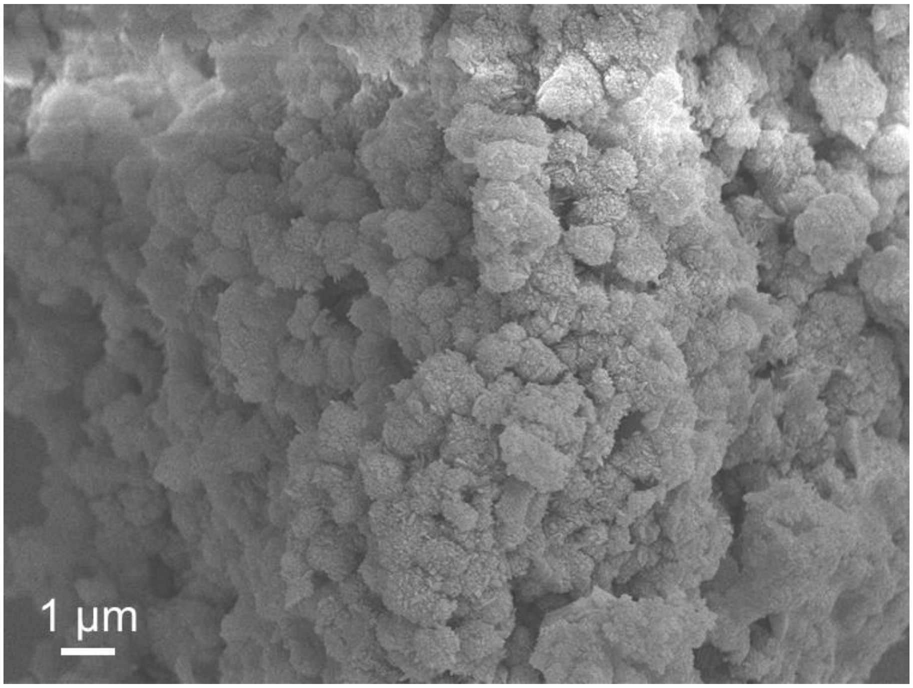

Figure S3. SEM image of TUS-39.

5. Thermogravimetric analysis (TGA)

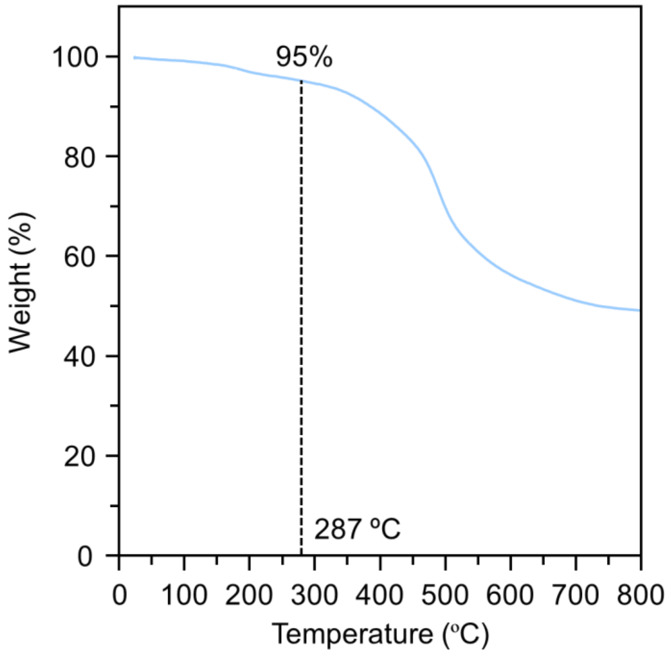

Figure S4. TGA curve of TUS-39 under N_2 atmosphere.

6. Chemical stability

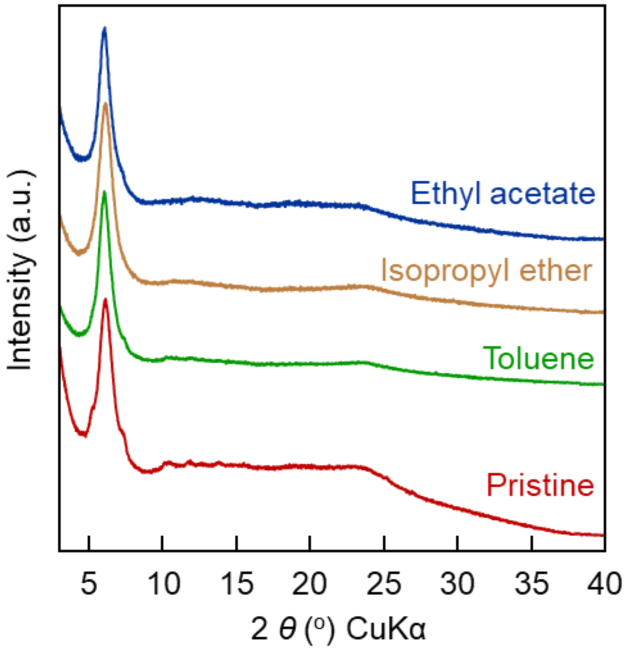

Figure S5. PXRD profiles of TUS-39 after immersing in different solvents for 24 hours.

7. BET surface area plot

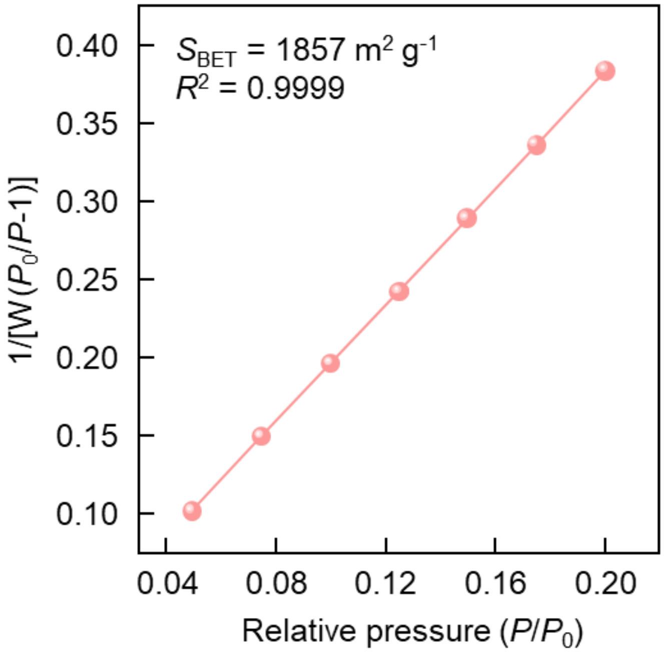

Figure S6. BET plot for TUS-39 calculated from the N_2 adsorption isotherms at 77 K. S_{BET} = 1857 m^2 g^{-1}, R^2 = 0.999.

8. Structural modeling and X-ray diffraction (XRD) analyses

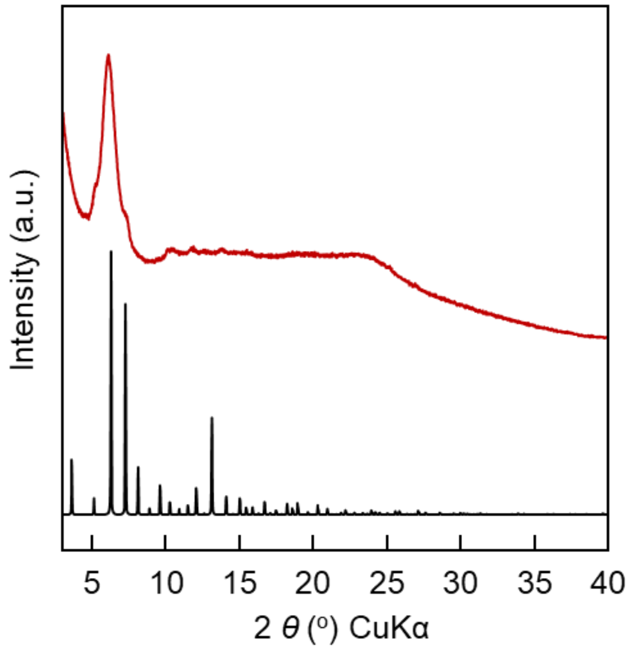

Figure S7. XRD patterns of TUS-39: experimental (red) and simulated (black) with 2-fold interpenetrated **the** topology.

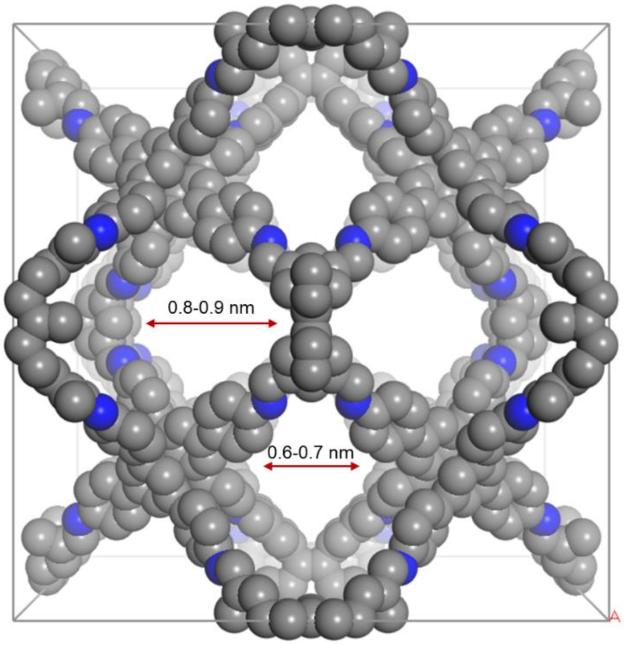

Figure S8. Space-filling model of TUS-39 adopting 2-fold interpenetrated **the** net with *Im*-*3* (No. 204) space group. C, gray; N, blue.

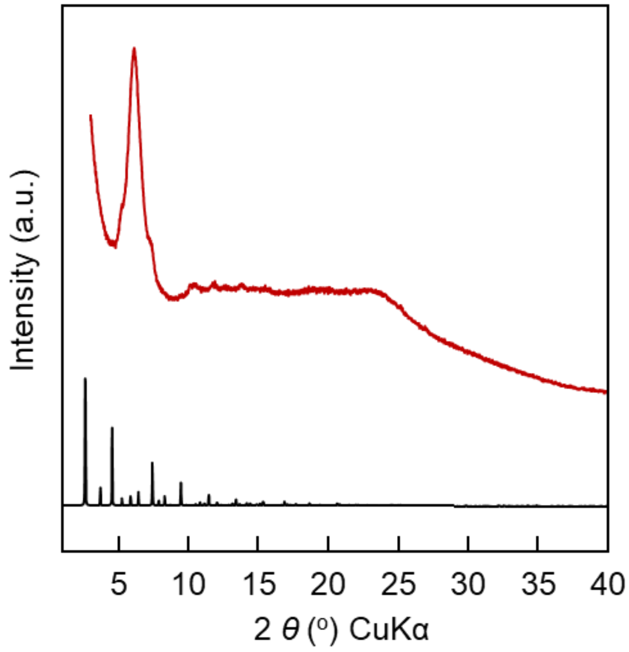

Figure S9. XRD patterns of TUS-39: experimental (red) and simulated (black) with non-interpenetrated **the** topology.

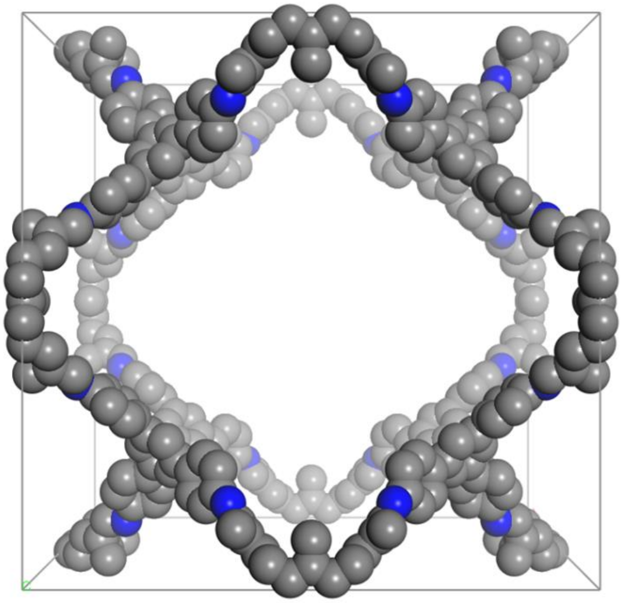

Figure S10. Space-filling model of TUS-39 adopting non-interpenetrated **the** net with *Pm*-*3* (No. 200) space group. C, gray; N, blue.

9. Immobilizing amano lipase PS onto/into COFs

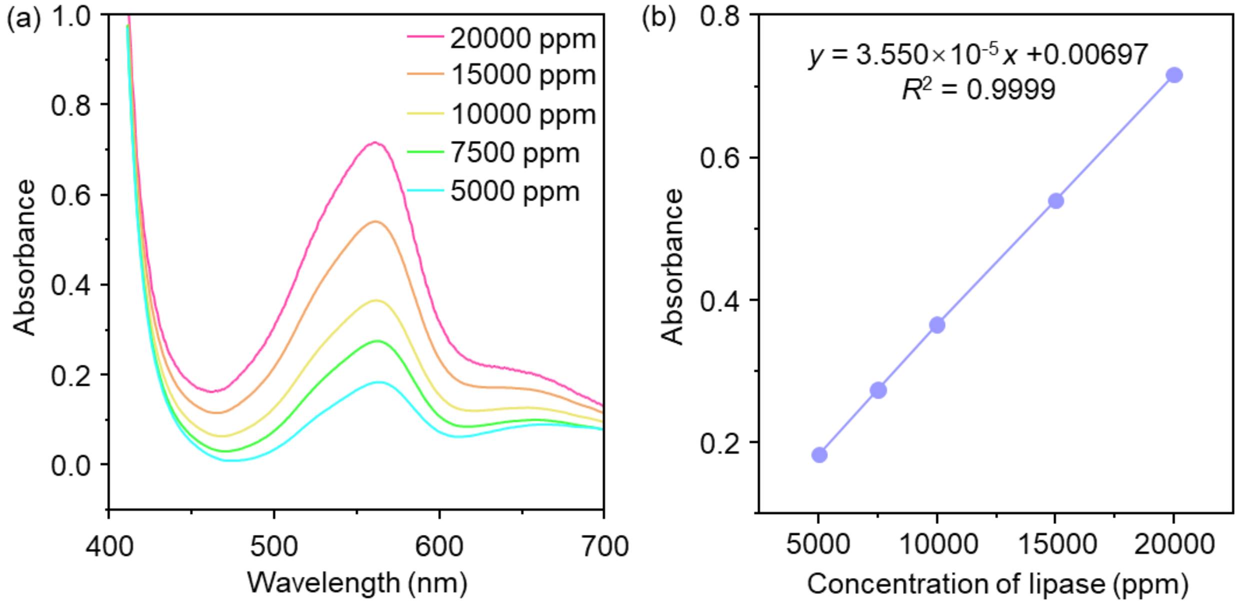

Figure S11. (a) UV/Vis spectra displaying the absorbance of different concentrations of lipase using BCA assays, and (b) standard curve derived from Figure S11a.

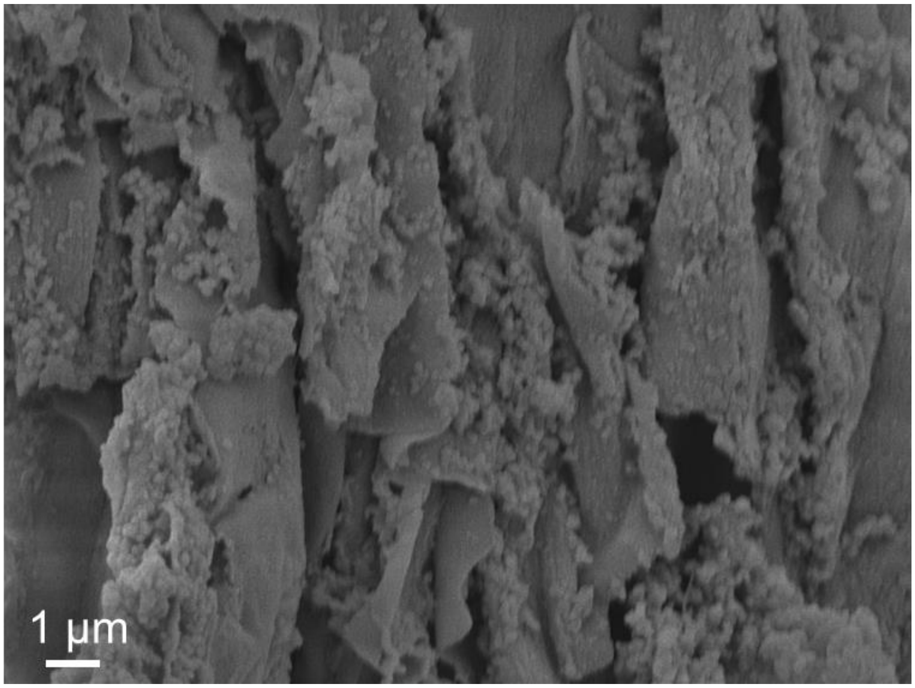

Figure S12. SEM image of lipase@TUS-39.

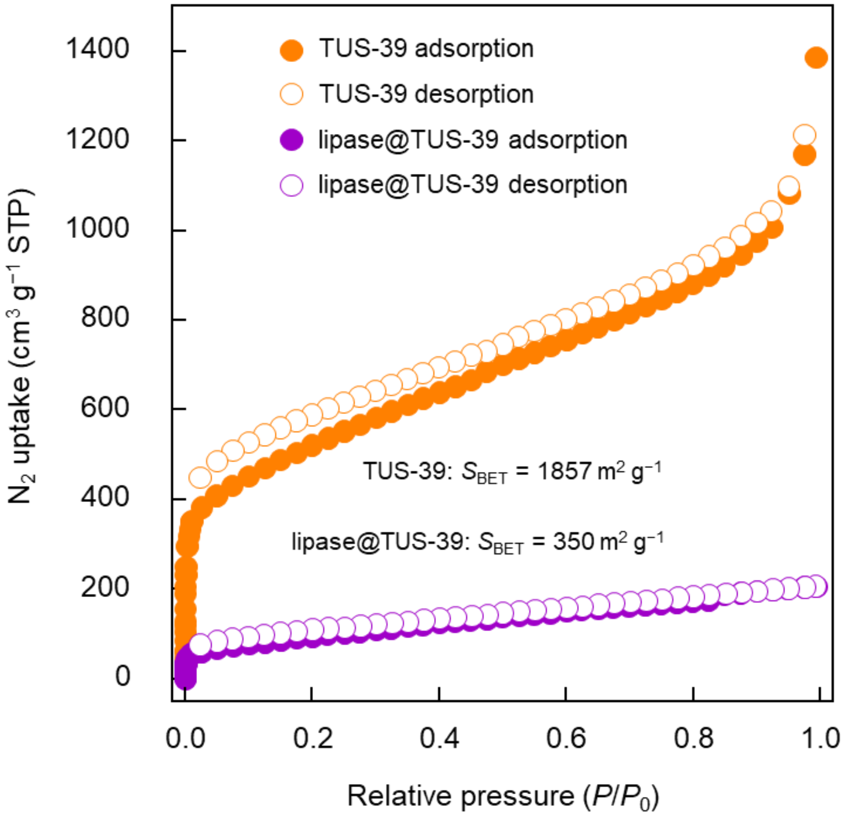

Figure S13. N_2 sorption isotherms of lipase@TUS-39 (purple) and TUS-39 (orange) at 77 K.

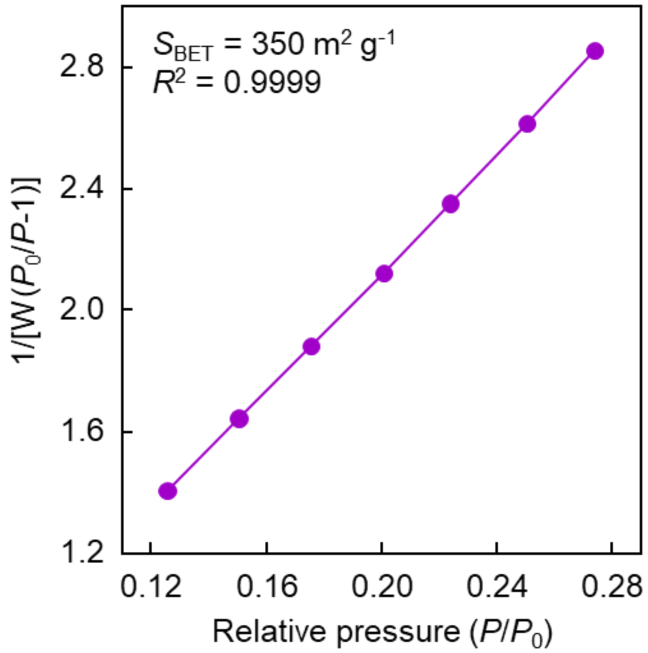

Figure S14. BET plot for lipase@TUS-39 calculated from the N_2 adsorption isotherms at 77 K. S_{BET} = 350 m^2 g^{-1}, R^2 = 0.999.

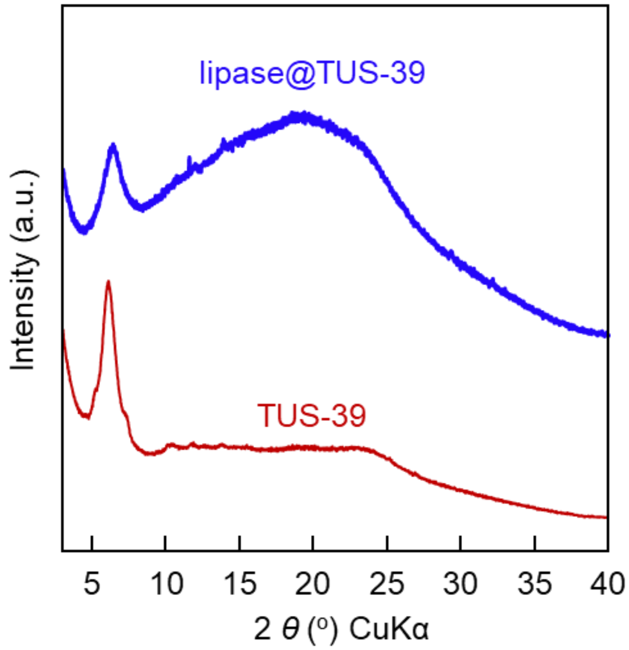

Figure S15. PXRD patterns of TUS-39 (red) and lipase@TUS-39 (blue).

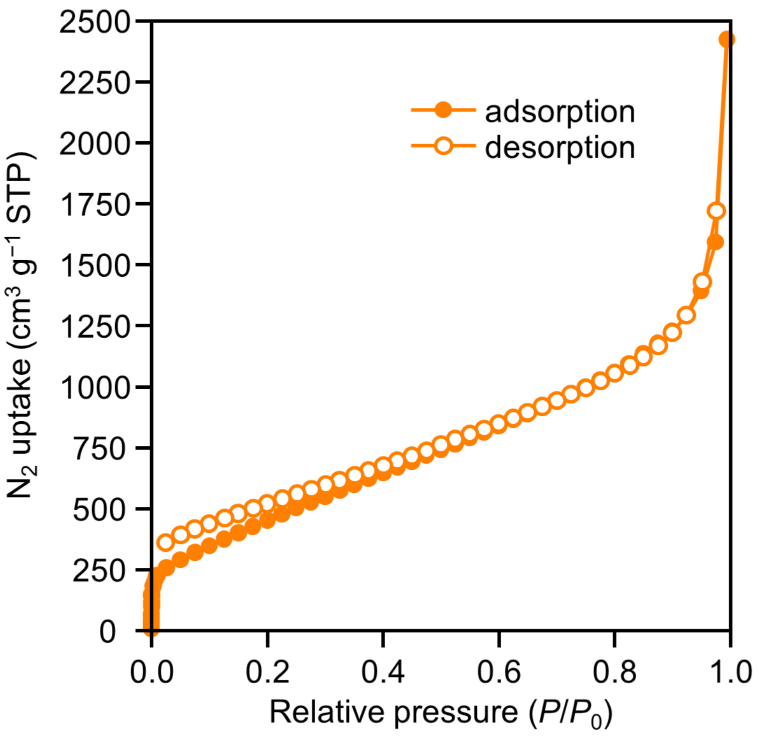

Figure S16. N_2 sorption isotherms of TUS-39 measured at 77 K after soaking for 6 h in buffer without enzyme.

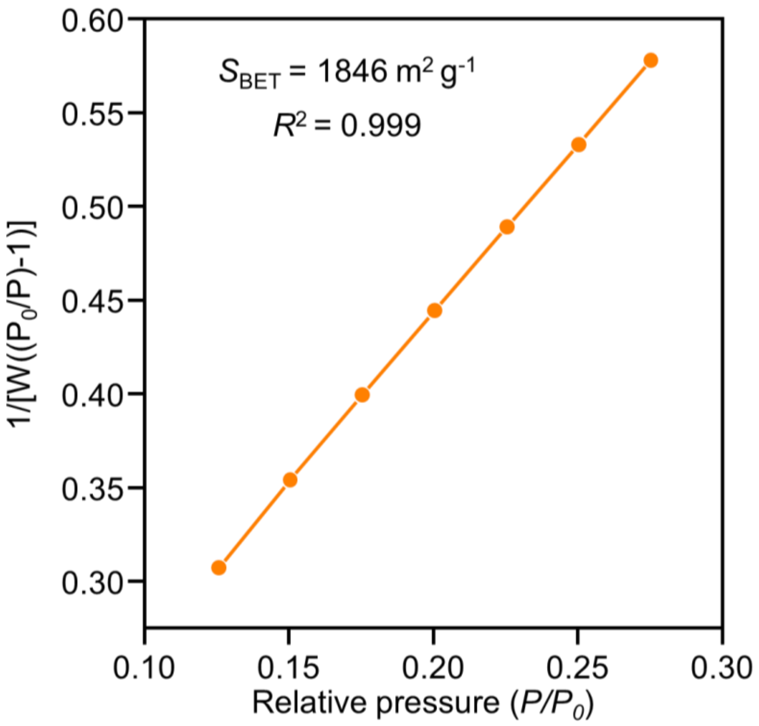

Figure S17. BET plot for TUS-39 after soaking for 6 h in buffer without enzyme, calculated from the N_2 adsorption isotherms at 77 K. S_{BET} = 1846 m^2 g^{-1}, R^2 = 0.999.

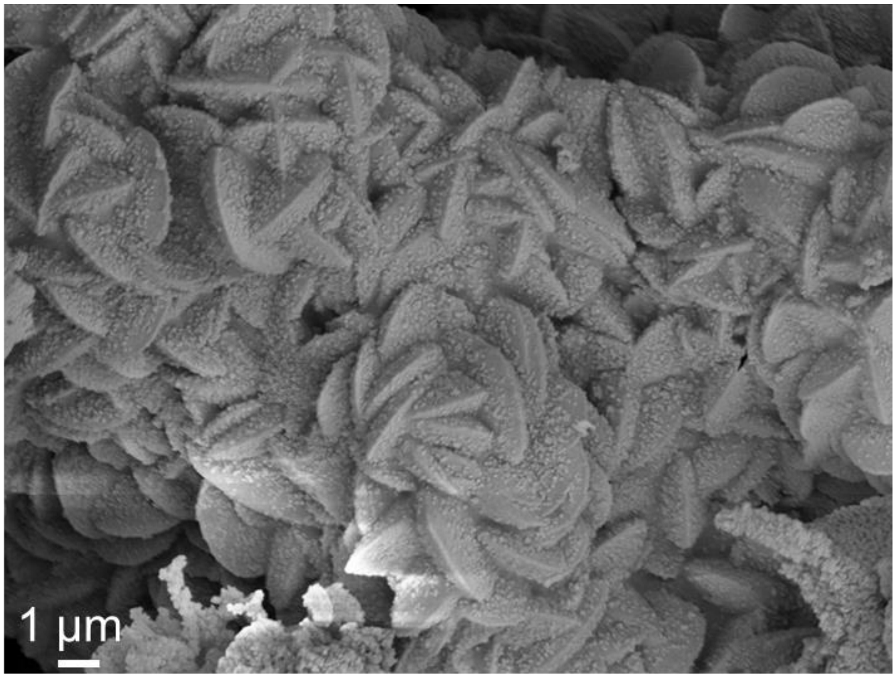

Figure S18. SEM image of TUS-64.

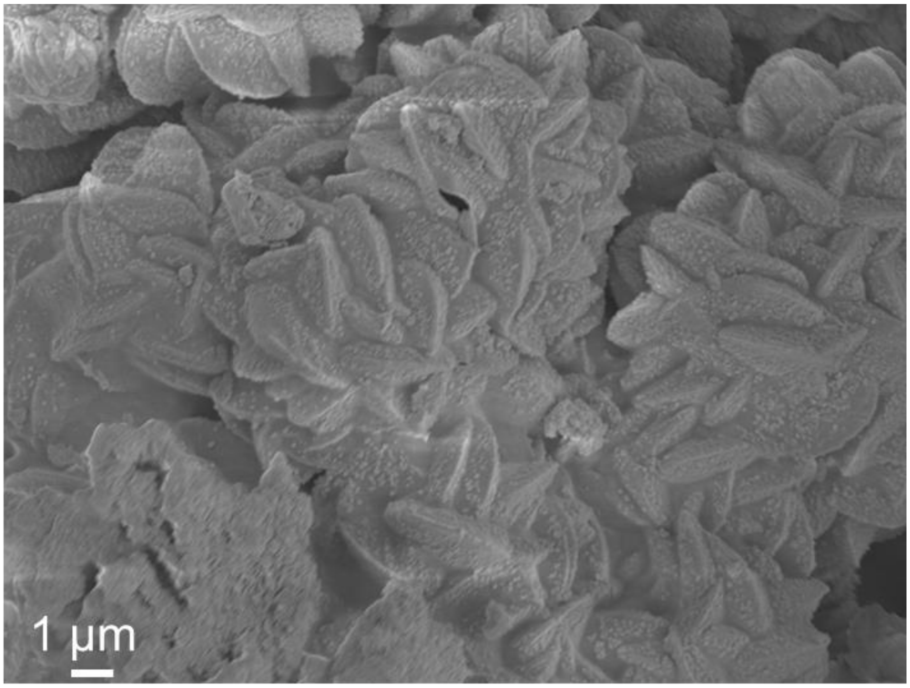

Figure S19. SEM image of lipase@TUS-64.

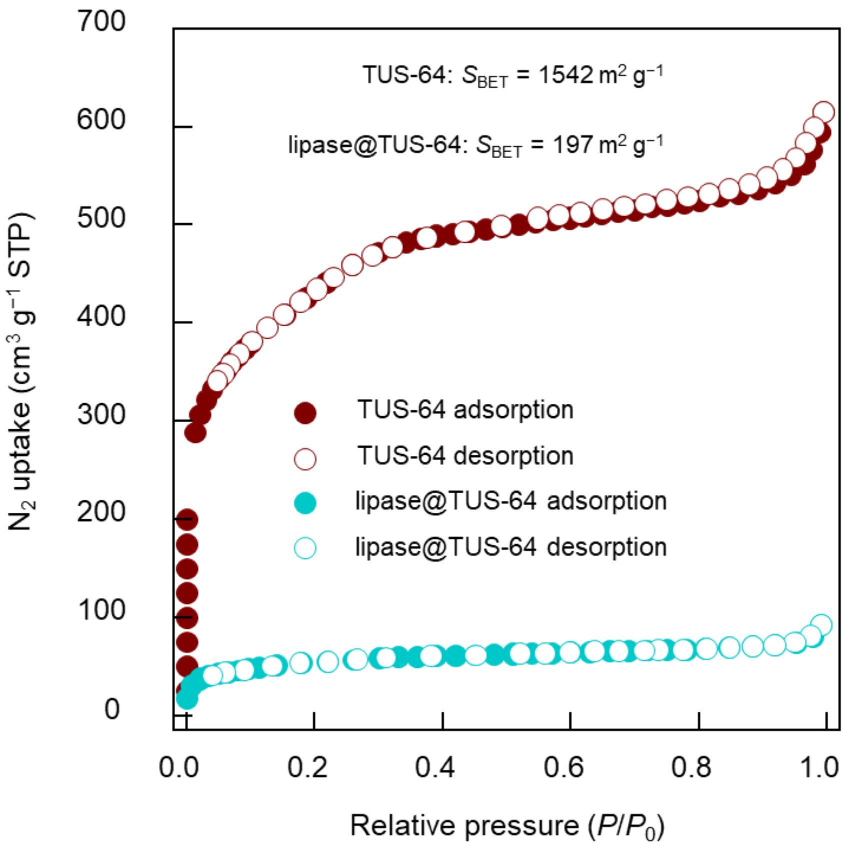

Figure S20. N_2 sorption isotherms of TUS-64 (brown) and lipase@TUS-64 (cyan) at 77 K.

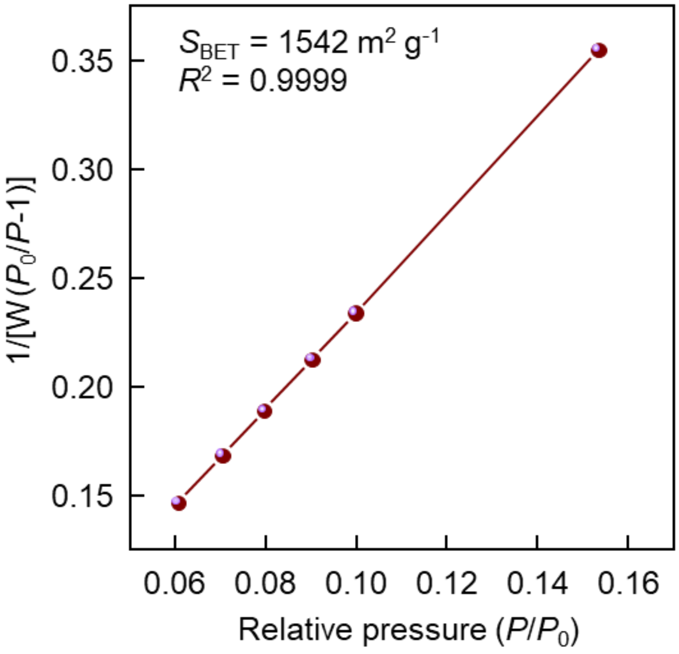

Figure S21. BET plot for TUS-64 calculated from the N_2 adsorption isotherms at 77 K. S_{BET} = 1542 m^2 g^{-1}, R^2 = 0.999.

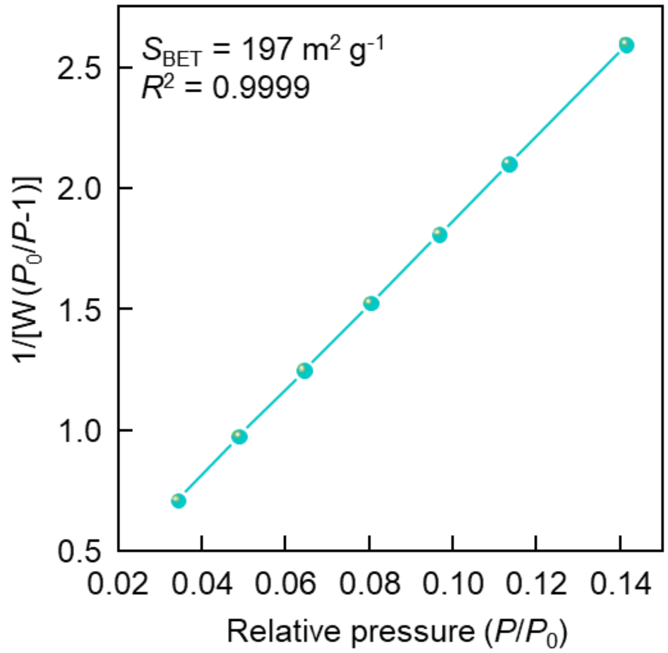

Figure S22. BET plot for lipase@TUS-64 calculated from the N_2 adsorption isotherms at 77 K. S_{BET} = 197 m^2 g^{-1}, R^2 = 0.999.

10. Lipase-catalyzed kinetic resolution of (*R*,*S*)-1-phenylethanol

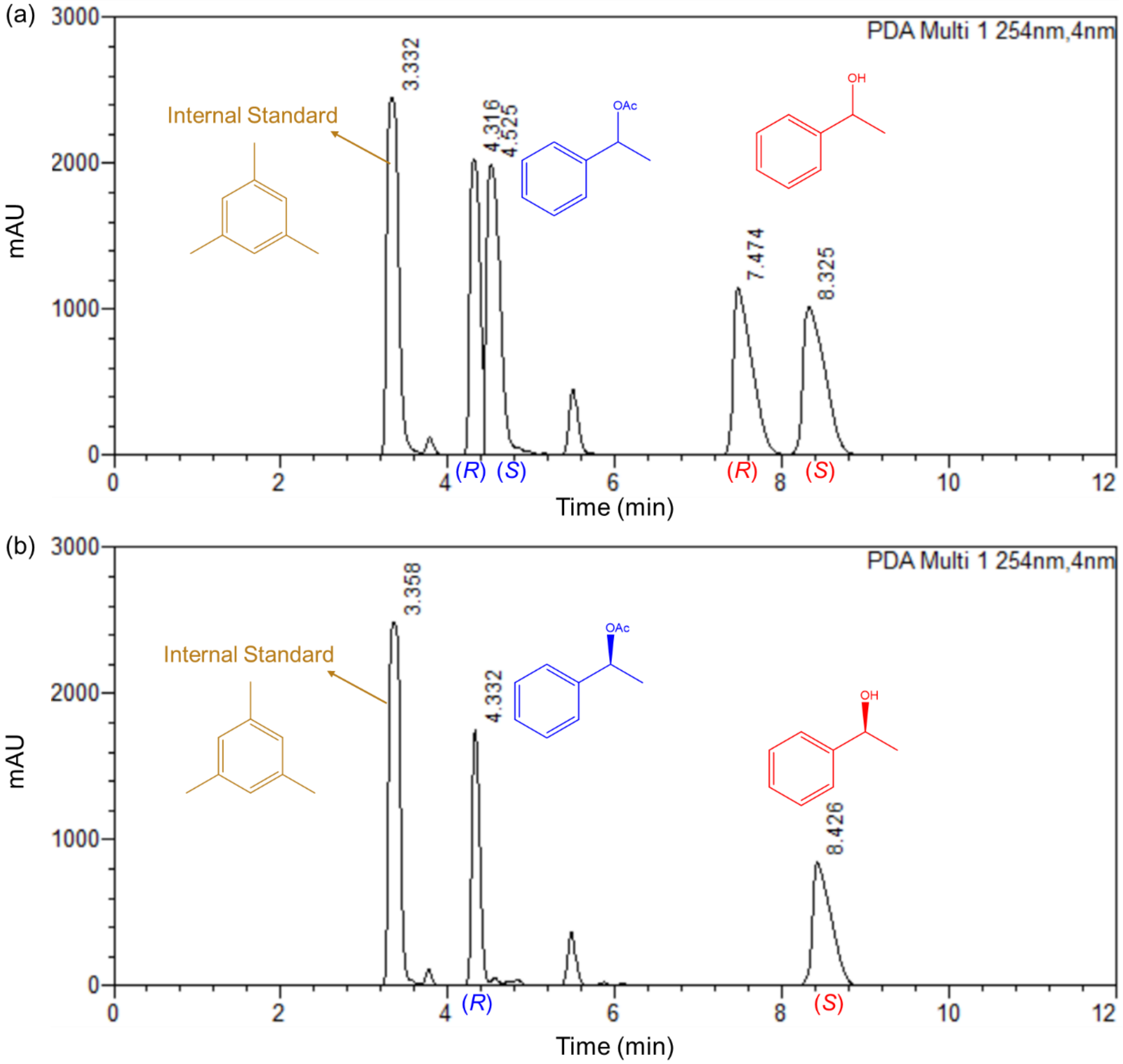

Figure S23. HPLC chromatograms of (a) (*R*,*S*)-1-phenylethyl acetate and (*R*,*S*)-1-phenylethanol, and (b) the solution obtained following the kinetic resolution of (*R*,*S*)-1-phenylethanol employing vinyl acetate as the acyl donor, *n*-hexane as the reaction medium and catalyzed by lipase@TUS-39.

11. FT-IR and circular dichroism (CD) characterization of lipase–TUS-39 interactions

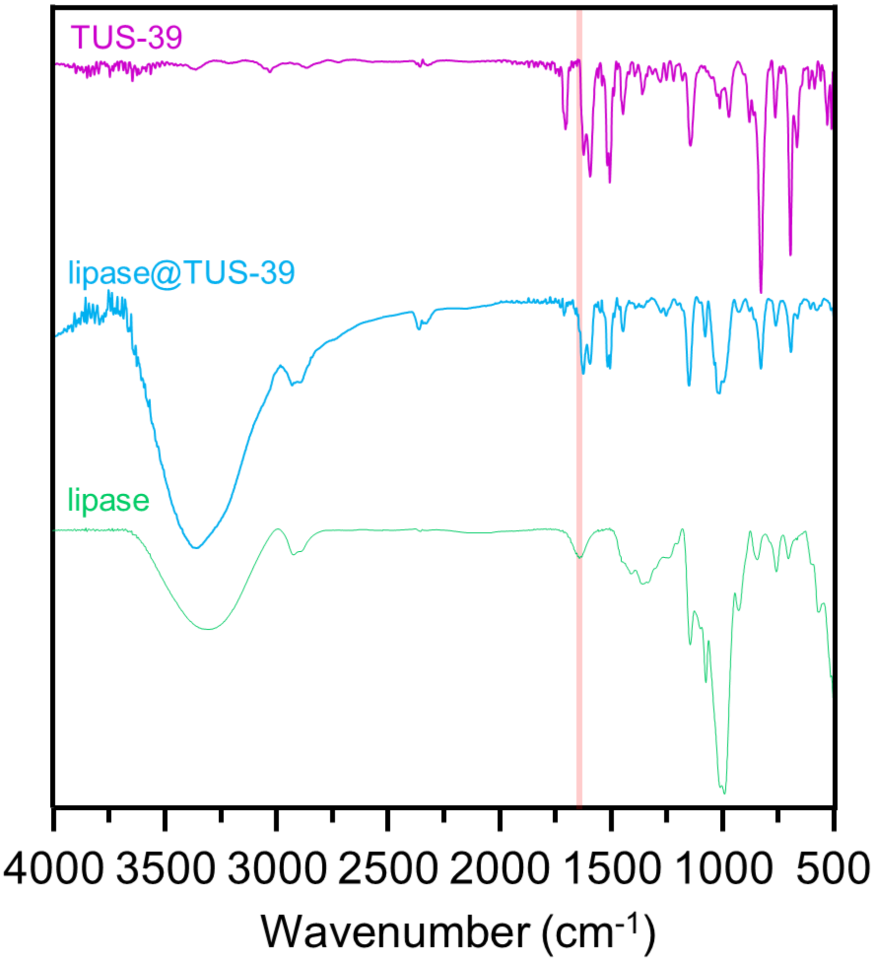

Figure S24. FT-IR spectra of lipase@TUS-39, lipase, and pristine TUS-39.

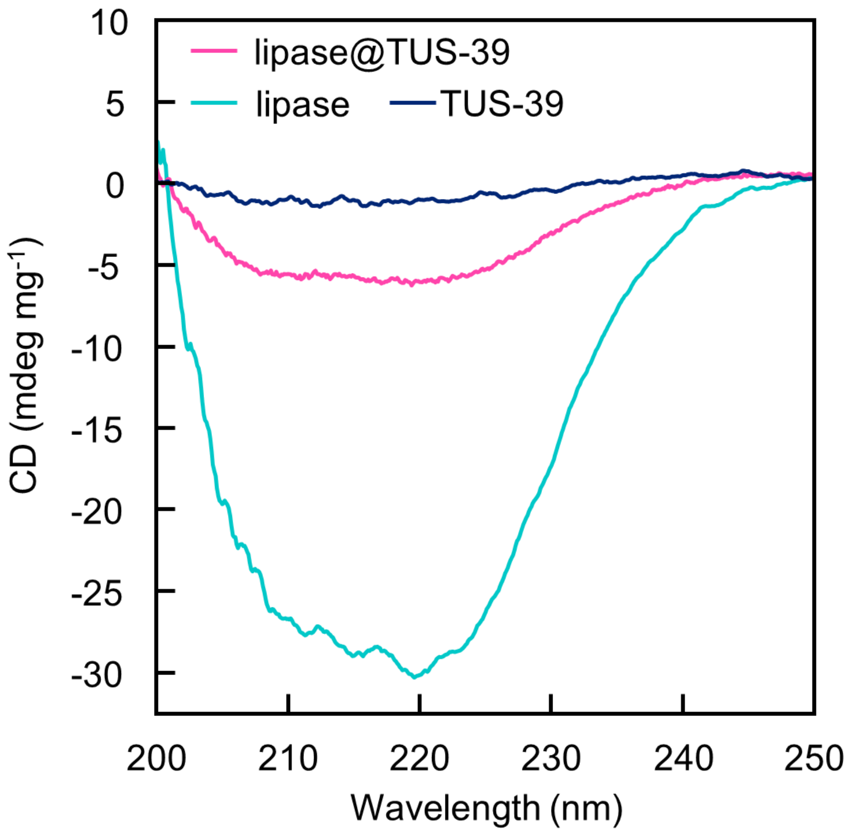

Figure S25. CD spectra of pristine TUS-39, free lipase, and lipase@TUS-39. TUS-39 exhibits a flat baseline, free lipase shows a pronounced negative ellipticity down to –30 mdeg mg^{-1}, while lipase@TUS-39 displays a shallower valley at –5 mdeg mg^{-1}, indicating that the enzyme largely retains its secondary structure after immobilization on the COF surface.

12. Molecular docking simulations

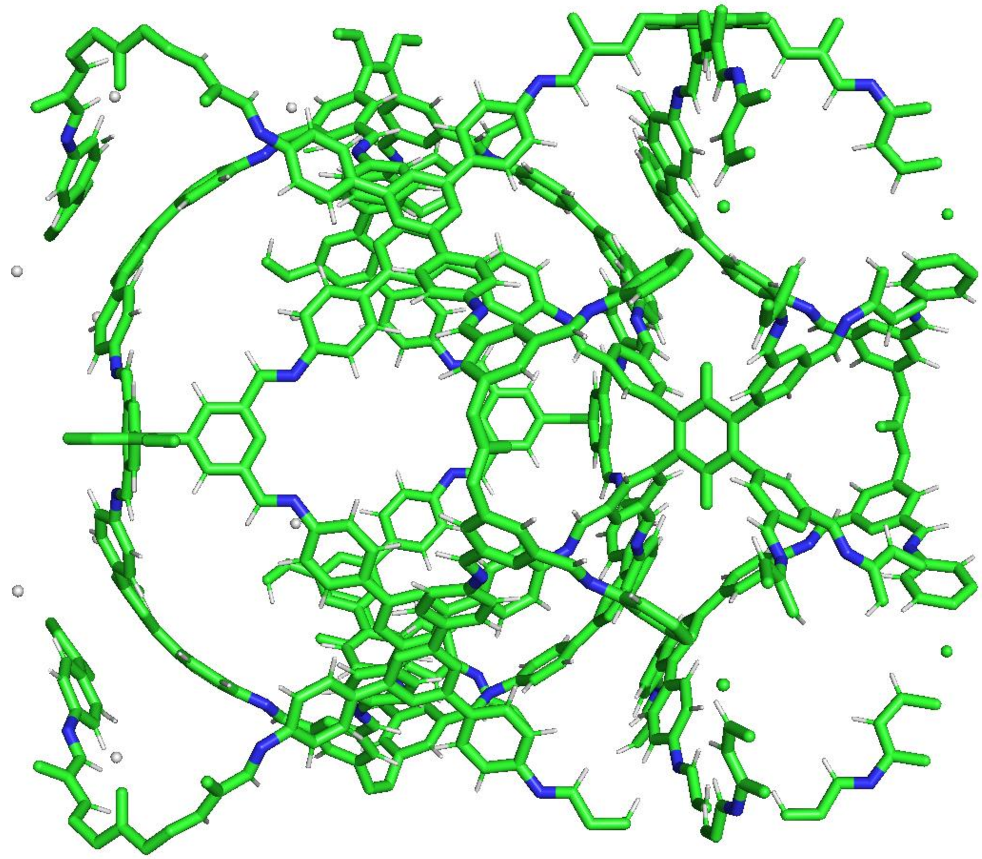

Figure S26. TUS-39 treated as the ligand.

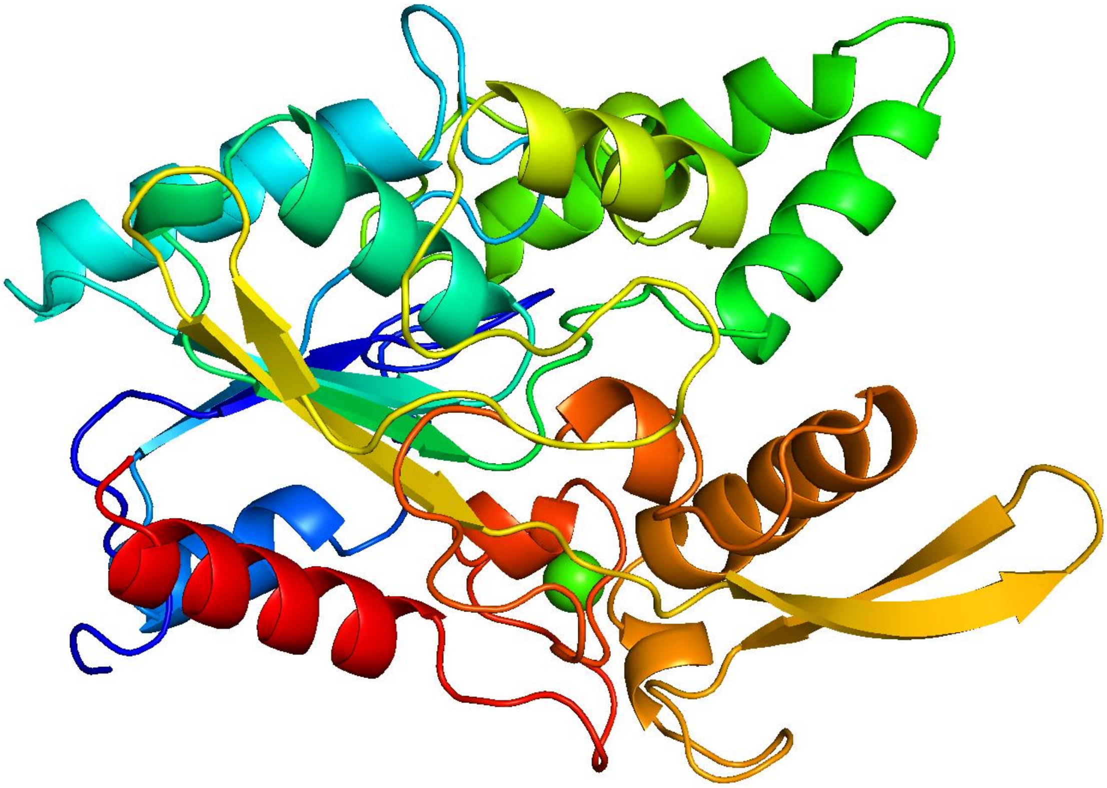

Figure S27. The crystallographic structure of lipase used as the receptor.

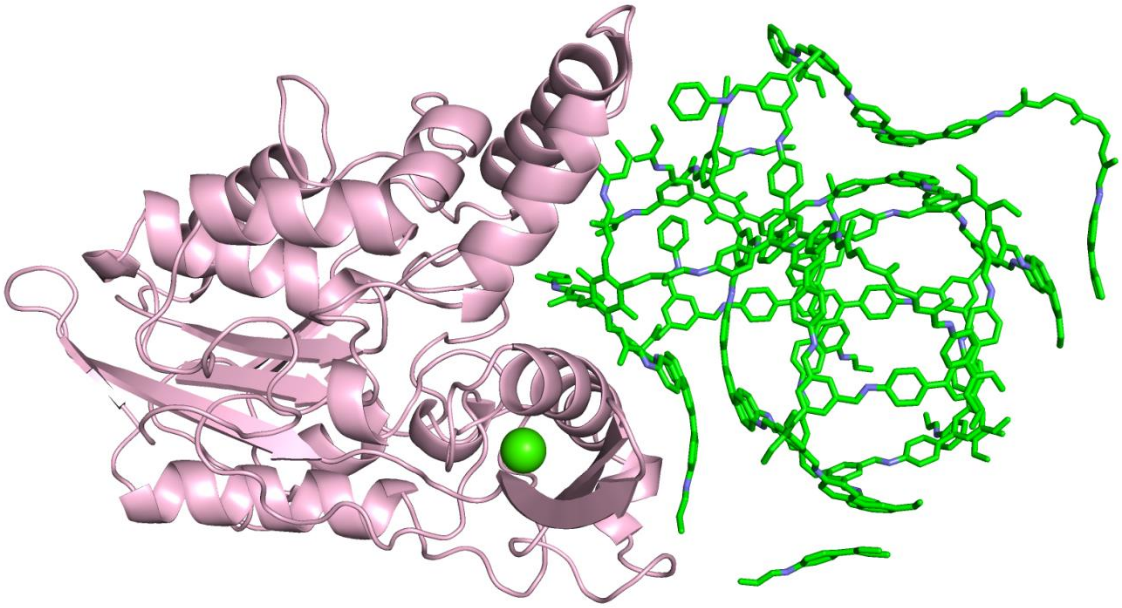

Figure S28. Representative docking conformations of TUS-39 within the active site of Amano lipase PS obtained through AutoDock simulations.

Table S1. Summary of bonding interactions identified from docking simulations between TUS-39 and lipase PS.

Name	Distance (Å)	Category	Type
lig - ILE139	4.08813	Hydrophobic	Alkyl
ligC36 - VAL235	5.32717	Hydrophobic	Alkyl
PHE142 - lig	4.92787	Hydrophobic	Pi-Alkyl
PHE146 - lig	5.08214	Hydrophobic	Pi-Alkyl
lig - PRO243	4.88405	Hydrophobic	Pi-Alkyl
lig - ALA240	4.59931	Hydrophobic	Pi-Alkyl

13. Unit cell information and fractional atomic coordinates

Table S2. Unit cell information and fractional atomic coordinates of TUS-39 calculated based on the 2-fold interpenetrated **the** net.

Space group		*Im*-3 (No. 204)	
Unit cell		$a = b = c = 34.4451$ Å, $\alpha = \beta = \gamma = 90^{o}$	
Pawley refinement		$R_p = 0.81\%$, $R_{wp} = 1.08\%$	
Atoms	x	y	z
C	0.12386	0.86392	0.69034
C	0.13999	0.83733	0.71657
C	0.08108	0.03541	0.62
C	0.09593	0.07169	0.63723
C	0.05518	0.0354	0.58858
C	0.17815	0.90713	0.70115
C	0.19434	0.88063	0.72716
C	0.16868	0.78892	0.77904
C	0.18937	0.82057	0.76784
C	0.17495	0.84585	0.73604
C	0.14261	0.89945	0.68308
N	0.1251	0.93019	0.6611
C	0.46443	0.02048	0
C	0.36486	0.09372	0
C	0.42757	0.04263	0
C	0.0408	0	0.5
C	0.91557	0	0.5

14. Supplementary references

(1) Jin, F.; Lin, E.; Wang, T.; Geng, S.; Wang, T. Liu, W.; Xiong, F.; Wang, Z.; Chen, Y.; Cheng, P.; Zhang, Z. *J. Am. Chem. Soc.* **2022**, *144*, 5643–5652.

(2) Bao, C.; Jin, M.; Lu, R.; Song, Z.; Yang, X.; Song, D.; Xu, T.; Liu, G.; Zhao, Y. *Tetrahedron* **2007**, *63*, 7443–7448.

(3) Bao, C. Y.; Lu, R.; Jin, M.; Xue, P. C.; Tan, C. H.; Xu, T. H.; Liu, G. F.; Zhao, Y. Y. *Chem.—Eur. J.* **2006**, *12*, 3287–3294.
